# High‐Dimensional Propensity Scores for Mitigating Confounding: Implementation Using Primary and Secondary Care Data in Hong Kong

**DOI:** 10.1002/pds.70326

**Published:** 2026-01-25

**Authors:** Edmund C. L. Cheung, Min Fan, Celine S. L. Chui, Angel Y. S. Wong, John Tazare

**Affiliations:** ^1^ Centre for Safe Medication Practice and Research, Department of Pharmacology and Pharmacy, Li Ka Shing Faculty of Medicine The University of Hong Kong Hong Kong China; ^2^ School of Nursing, Li Ka Shing Faculty of Medicine The University of Hong Kong Hong Kong China; ^3^ School of Public Health, Li Ka Shing Faculty of Medicine The University of Hong Kong Hong Kong China; ^4^ Faculty of Epidemiology and Population Health London School of Hygiene and Tropical Medicine London UK

**Keywords:** antihypertensive drugs, confounding, dementia, high‐dimensional propensity score, observational studies

## Abstract

**Purpose:**

Confounding is a key concern in observational studies using healthcare databases. The high‐dimensional propensity score (HDPS) algorithm is an approach for generating and prioritising proxy variables, leveraging all available information in a database to mitigate residual confounding. This study aims to implement HDPS approaches in a novel setting using primary and secondary data available from Hong Kong (HK).

**Methods:**

Using data from HK, we implemented HDPS in a cohort study investigating the use of different antihypertensive drug classes and incident dementia risk. The top 250 HDPS covariates were included in inverse probability of treatment weighting in addition to investigator‐specified variables. Diagnostics evaluated the performance of the HDPS. Sensitivity analyses included varying the number of HDPS covariates and removing potentially influential or inappropriate covariates.

**Results:**

434 506 new‐users of antihypertensives were included. With a traditional PS approach, no evidence for an association was observed for each antihypertensive comparison. After HDPS implementation, the estimate for beta‐blockers shifted from no evidence (Hazard ratio (HR): 0.93, 95% confidence interval (CI): 0.86–1.02) to moderate evidence of a reduced hazard of incident dementia compared to angiotensin‐converting enzyme inhibitors (HR: 0.90, 95% CI: 0.82–0.98). A greater overall covariate balance between comparison groups was achieved after the inclusion of HDPS covariates and potential frailty markers were identified as influential.

**Conclusions:**

We successfully implemented the HDPS in HK data, observing improved covariate balance across a wider set of potential confounders. HDPS also identified possible database‐specific frailty markers which could be considered more widely when specifying adjustment variables in this setting.

## Key Points

- The aim of the study was to implement the high‐dimensional propensity score (HDPS) algorithm, a method for mitigating bias due to confounding in observational studies, in a novel setting using primary and secondary data from Hong Kong.
- We compared the results of the case study before and after application of HDPS, evaluated the performance using diagnostics, and developed an R package for HDPS implementation in any database.
- HDPS analyses are encouraged in future observational studies using electronic healthcare databases to mitigate confounding and identify potentially influential covariates that may be neglected when using clinical or epidemiological knowledge solely for variable selection.

## Plain Language Summary

Confounding is a key concern in observational studies using healthcare databases. The HDPS is a method that can leverage all available information in a database to mitigate residual confounding. This study aims to implement HDPS approaches in a novel setting using primary and secondary data available from Hong Kong (HK). Using data from HK, we applied HDPS in a study comparing the use of different antihypertensive drug classes and dementia risk. We also used diagnostics to evaluate the performance of the HDPS. In the results with a traditional propensity score approach, no evidence for an association was observed for each antihypertensive comparison. After HDPS implementation, the estimate for beta‐blockers shifted from no evidence to moderate evidence of a reduced risk of dementia compared to angiotensin‐converting enzyme inhibitors. A greater balance of variables between comparison groups was achieved with HDPS, indicating improved performance compared with the traditional propensity score approach, and additional markers with potential confounding were identified as influential. We also developed a statistical package to ease its implementation in future studies. In conclusion, we successfully implemented the HDPS in HK data and encourage its uptake in observational studies using healthcare data.

## 1 Introduction

Bias due to confounding is a key concern in observational studies and failure to adequately mitigate this can lead to invalid causal treatment effects [1]. For a given study, confounding should be considered in both the study design phase (e.g., defining appropriate study inclusion criteria, using active treatment comparators where possible), and the analysis phase (e.g., propensity score (PS) methods, multivariable adjustment) [2].

In the analysis phase, PS methods are increasingly used in observational studies aiming to estimate effects of treatment [3]. The PS is the probability of receiving treatment given observed baseline characteristics and requires a two‐stage process. The first is estimating the PS, usually via logistic regression, and the second is incorporating the PSs to estimate treatment effects, commonly via matching and weighting methods [4, 5]. The PS is a summary measure and condenses information from a large number of variables, included in the PS model, into a single estimate. The variables included are traditionally selected using a combination of clinical and epidemiological knowledge. The high‐dimensional propensity score (HDPS) algorithm, originally developed in claims data, was introduced as a data‐driven approach to empirically identify, prioritise, and select covariates in large healthcare databases, augmenting the PS by adding additional covariates to improve confounding control. The principle underlying the HDPS is that the large amount of data contained in these databases can contain information related to unmeasured confounding and can act as proxy variables. The HDPS conceptualises information as proxies for concepts that may contribute to residual confounding but be poorly measured, for example, frailty [6, 7]. This approach has been shown to be an effective method for mitigating confounding and can improve covariate balance when compared to the traditional PS approach in comparative studies [8, 9]. The HDPS can also potentially identify covariates, specific to the database under investigation, that may be omitted in approaches based on clinical knowledge alone.

HDPS has been applied across a variety of different settings including insurance claims data and electronic health records; however, there is a lack of applications using routine clinical data in settings where both primary and secondary care records are available (e.g., Clinical Data Analysis and Reporting System in Hong Kong) [10, 11]. Furthermore, there is currently no integrated package to conduct HDPS analysis in R [12, 13], which would simplify the application of HDPS approaches and potentially encourage further uptake.

In this study, we aimed to demonstrate the implementation of HDPS approaches in routinely collected clinical data in Hong Kong (HK). We evaluated the performance of HDPS in the context of a case study comparing exposure to different antihypertensive drug classes and developed a versatile R package to aid application of HDPS approaches, more generally.

## 2 High Dimensional Propensity Scores

Implementation of HDPS in any context requires consideration of the steps proposed by Schneeweiss et al. and related investigators' decisions [6]. This includes specification of details and choices surrounding data dimension specification, candidate covariate identification, recurrence assessment, covariate prioritisation, covariate selection, and treatment effect estimation. A summary of the HDPS procedure is provided in Table 1.

**TABLE 1:** Summary of high‐dimensional propensity score (HDPS) steps and application to Hong Kong data.

Step number	Step name	Description	Application to Hong Kong data	Software implementation with R package
1	Data dimension specification	– Information in healthcare databases is separated into a number of dimensions each representing an aspect of care within the healthcare system. – A covariate assessment window in which to identify information in these dimensions is required.	– Three data dimensions were formed separating information on: diagnoses (in‐patient and outpatient), prescriptions, and laboratory measurements. – Covariate assessment windows: entire medical history for diagnoses, 90 days for prescription and 1 year for laboratory measurements prior to cohort entry (to align with the original study).	Data for each data dimension should be separated and organised in a long format: one column for unique patient identifiers and one column for codes representing the relative aspect of care, e.g., diagnosis code. Patients with multiple records should appear in multiple rows. Example: ‘data(dx)’ for an example diagnosis dataset and ‘data(master)’ for an example master sheet with exposure and outcome information, both included with our ‘hdps’ package.
2	Candidate feature identification	– Given available information, candidate features are identified. – Large healthcare databases commonly store healthcare data using medical or product coding systems to classify diseases, medications, and other aspects of care. – To reduce the potential dimensionality of features, often some form of aggregation is usually applied (e.g., truncating codes to first 3‐digits in a hierarchical coding system). – Additionally, a prevalence filter may be applied. – Features that are close proxies of the exposure or outcome are removed to avoid circular reasoning	The granularity level set for the coding systems in Hong Kong HA data is described here. – Diagnoses data dimensions: *Outpatient diagnoses*: the International Classification of Primary Care (ICPC) and International Classification of Diseases (ICD‐9) *In‐patient diagnoses*: the same coding systems were used. Both are hierarchical coding systems with 3‐character ICPC and 3‐digit ICD‐9 codes being sufficient to represent distinct diagnoses. – Prescription data dimension: the British National Formulary (BNF) coding system was used with granularity set at the chapter level. – Laboratory measurements data dimension: local codes that were developed as unique numbers specific to each type of measurement in this particular dataset were used. There were ~800 total candidate covariates generated from all three dimensions, no prevalence filter was applied, and close proxies of antihypertensive exposure and dementia diagnosis were removed. All features were considered as binary covariates (presence/absence of feature regardless of prescription dose/laboratory measurement result).	This can be performed using the ‘identify_candidates’ function from our ‘hdps’ package. Example: identify_candidates(dt = dx, id = ‘pid’, code = ‘icd9code’, type = ‘dx’, *n* = 200, min_patients = 10) Arguments: dt = data table with patient and covariate information id = column name for patient identifier code = column name for codes (e.g., diagnosis codes, drug codes, etc.) type = prefix for output (e.g., dx, rx, px) *n* = maximum number of candidates to return min_ patients = minimum patients required per covariate for prevalence filtering
3	Feature recurrence assessment	– HDPS assumes a relationship between the frequency of a feature (code) appears within the baseline window and the underlying health status of individuals and/or health seeking behaviour. – The recurrence of each code in each individual is therefore measured in HDPS. This informs the generation of three binary HDPS covariates for each code.	– Consistent with the original HDPS proposal, recurrence cut‐offs for pre‐exposure features were based on features being recorded: ≥one time, ≥median times, and≥upper quartile number of times within the corresponding covariate assessment windows.	This can be performed using the ‘assess_recurrence’ function from our ‘hdps’ package. Example: assess_recurrence(dt = candidates$data, id = ‘'pid’, code = ‘code’, type = ‘dx’, rank = Inf) Arguments: dt = data table generated from step 2 id = column name for patient identifier code = column name for codes type = prefix for output (e.g., dx, rx, px) rank = maximum number of covariates to include
4	Covariate prioritisation	– The generated HDPS covariates are then prioritised, commonly using Bross formula‐based scoring variables based on the association between covariate and outcome, and covariate and exposure.	– HDPS covariates were prioritised using Bross formula‐based scoring variables. When there were few events in each exposure group, the zero‐cell correction was applied by adding 0.1 to all cells if there was any cell with a value of 0 in the 2 × 2 relative risk tables to allow all HDPS covariates to be considered for inclusion in the prioritisation step.	This can be performed using the ‘prioritise’ function from our ‘hdps’ package after merging the output from step 3 with the master sheet. Example: merge(recurrence, master, by = ‘pid’, all.x = TRUE) prioritise(dt = cohort_data, id = ‘pid’, expo = ‘exposure’, outc = ‘outcome’, correction = TRUE) Arguments: dt = data table after merge id = column name for patient identifier expo = column name for exposure outc = column name for outcome correction = application of zero‐cell correction
5	Covariate selection	– The top N number of covariates, specified by investigators, are then taken forward to be included in the propensity score model.	– The top 250 HDPS covariates were taken forward to be included in the propensity score model.	Select the top N number of covariates from the output in step 4, ranked based on the ‘absLogBias’ column from the step 4 output (higher absLogBias = higher ranked).
6	Propensity score estimation	– The prioritised HDPS covariates are added to the propensity score estimation on top of any investigator pre‐defined variables.	– PS were estimated using logistic regression and the top 250 HDPS covariates were added to the 32 pre‐defined variables.	Add the top N covariates to pre‐defined variables and execute propensity score method, e.g., ‘weightit’ function from the ‘WeightIt’ package.
7	Outcome estimation	– The outcome model is estimated with the desired propensity score method applied.	– The outcome model was Cox proportional hazards regression with inverse probability treatment weighting.	Generate the outcome estimates with the propensity score method applied, e.g., ‘coxph’ function from the ‘survival’ package.

First, we split information into data dimensions, separating different aspects of the healthcare database (e.g., diagnoses, prescriptions, and procedures) and capturing data within a specified covariate assessment window (e.g., 1 year prior to cohort entry). During this step, codes will often be aggregated to a higher granularity, depending on coding system (e.g., truncating to 3‐digit International Classification of Diseases, 9th edition (ICD‐9) codes for diagnoses), and candidate features based on this granularity are identified from each data dimension. Second, a prevalence filter may be applied selecting the codes with the highest prevalence from each dimension (i.e., the proportion of patients in the cohort with at least one recording of the code) [6]. However, there is debate surrounding the utility of the prevalence filter [14] and this may be avoided when computational burden is not thought to be a concern. Third, code recurrence is assessed for the candidate features with binary covariates generated based on frequency cut‐offs. Fourth, these generated HDPS covariates are prioritised and ranked by their potential for confounding, commonly via the Bross formula [15]. Next, a subset of the top Bross‐ranked HDPS covariates are selected and included in the PS model in addition to any investigator pre‐defined variables. Finally, the outcome model is estimated with the desired PS method applied [3].

## 3 Methods

### 3.1 Data Source

The data was requested from the HK Hospital Authority (HA), a statutory body that manages HK's public healthcare services including primary, secondary, and tertiary services. The electronic health records captured from HA services are routinely collected clinical data in outpatient clinics and hospital settings. The records are de‐identified and include patients' demographics, diagnoses, prescriptions, laboratory measurements, admission and discharge information from accident and emergency, in‐patient, out‐patient, specialist, and pharmacy settings. This database has been validated in previous studies with high positive predictive values of diagnoses such as stroke and myocardial infarction [16, 17].

### 3.2 Design

We used HK data from a recent multinational cohort study investigating the association between antihypertensive use and the risk of incident dementia [18]. The study included longitudinal data from patients who received a blood pressure measurement in HA from 1 January 2005 to 31 December 2019. New‐users of antihypertensive medication were identified and new‐users of angiotensin‐II receptor blockers (ARB), beta‐blockers, calcium channel blockers (CCB), diuretics, and two or more concurrent classes of antihypertensives (Combination) were separately compared to new‐users of angiotensin‐converting enzyme inhibitors (ACEI). Patients were followed up from antihypertensive initiation and censored at the earliest of dementia diagnosis, death, or study end date (31st December 2020). The full details of the study design are described in the original study article [18].

This case study was chosen given anticipated residual confounding by indication due to hard‐to‐measure differences across antihypertensive drug classes. Such differences include frailty affecting the risk of dementia [19], as well as mechanisms of influence between antihypertensive drug class and dementia that have yet to be identified [20].

### 3.3 Statistical Analysis

The original study estimated PS using logistic regression and derived inverse probability of treatment weights (IPTW). Stabilised weights trimmed at the 99th percentile were used to reduce variance in the effect estimate. Hazard ratios (HR) and 95% confidence intervals (CI) were estimated using Cox models incorporating the IPTWs to compare the hazards of incident dementia between individual antihypertensive classes and ACEIs.

All HDPS analyses defined dimensions separating diagnoses, prescriptions, and laboratory measurements and no prevalence filter was applied. As with other applications of HDPS to routinely collected EHR data, the covariate assessment period for clinical diagnoses covered the entire patient history prior to cohort entry (as described in Table 1) [14, 21]. Generated HDPS covariates were prioritised using the Bross formula and, in primary analyses, the top 250 HDPS covariates supplemented the existing 32 investigator pre‐specified variables in the PS model.

In sensitivity analyses, we varied the number of HDPS covariates (top 50, 100, 150, 200 covariates) to investigate the robustness of results to the number of Bross‐ranked HDPS covariates included. We also investigated the impact of potentially influential covariates by (i) repeating the analyses only including the top 10 Bross‐ranked HDPS covariates and (ii) by removing covariates that empirically are strongly related to the exposure but not the outcome (i.e., empirically behaving like instrumental variables (IVs)). This was achieved by removing covariates meeting the following criteria: |logRR_CE_| > 1.5 and |logRR_CD_| < 0.5 (where RR_CE_ is the crude risk ratio for covariate‐exposure association and RR_CD_ is the crude risk ratio for covariate‐outcome association) [22].

### 3.4 Diagnostics of HDPS Covariates

The empirical properties of the HDPS covariates were assessed by describing the covariate‐outcome and covariate‐exposure associations. We compared the estimated PS distributions before (i.e., using pre‐defined covariates only) and after inclusion of the HDPS covariates. Covariate balance was also assessed using absolute standardised differences across all variables before and after incorporating the HDPS.

### 3.5 Software

All analyses were conducted in R version 4.3.0 (R Foundation for Statistical Computing, Vienna, Austria). PS weighting was performed using the ‘WeightIt’ package in R [23]. We developed an R package for easy implementation of the general HDPS procedure that can be transported to other settings [24]. This package is available via GitHub, with instructions for installation, corresponding documentation, and example code snippets, see [https://github.com/Cainefm/hdps]. Compared to existing R packages for conducting HDPS analyses including lendle/hdps [12] and technOslerphile/autoCovariateSelection [13], our package (cainefm/hdps) offers several improvements in terms of data manipulation for large datasets, integration of key steps of the process, and provision of simulated data. Compared to the existing packages, data manipulation is more efficient, especially for large samples, which is common in large observational studies. To demonstrate this, we conducted a timed comparison of the core steps of the HDPS process between our R package and other existing packages. As shown in Figure S1, our package was consistently more efficient when executing functions with the same purpose for samples up to 10 000 individuals, with 50%–127% faster times and 28%–43% less memory usage compared to the existing packages available. Advantages of our package also include: provision of simulated data for demonstrating implementation of the functions and testing, integration of diagnostic tools for HDPS analyses [10], and an in‐package interactive application with a user‐friendly interface. Our package also includes the option to correct for rare outcomes using the zero‐cell correction [25]. A versioned release of our R package at the time of article writing has been attached as Supporting Information S2.

## 4 Results

Baseline characteristics of the cohort are summarised in Table 2. 434 506 new‐users of antihypertensives were included with a mean age (standard deviation) of 61.8 (11.7) years at initiation. The mean age was highest in diuretics (66.3) and lowest in beta‐blockers (59.1). The median follow‐up ranged from 2.8 years in new‐users of angiotensin‐II receptor blockers (ARB) to 8.3 in new‐users of ACEI. Compared to other treatment groups, there was a higher prevalence of diabetes in the ACEI (48.7%) and ARB initiators (37.1%); with a corresponding pattern observed in non‐insulin antidiabetic drug usage.

**TABLE 2:** Baseline characteristics of new users of antihypertensives by class.

Baseline characteristics	All classes *N* = 434 506	ACEI *N* = 64 470 (14.2%)	ARB *N* = 8992 (2.0%)	Beta‐blocker *N* = 64 785 (14.3%)	CCB *N* = 246 423 (54.4%)	Diuretic *N* = 29 963 (6.6%)	Combination *N* = 19 873 (4.4%)
Age (mean, SD)	61.8 (11.7)	60.2 (11.2)	61.5 (11.0)	59.1 (11.7)	62.3 (11.5)	66.3 (13.0)	62.3 (11.3)
Age group (*N*, %)	—	—	—	—	—	—	—
40–49	65 541 (15.1)	11 299 (17.5)	1271 (14.1)	14 497 (22.4)	32 736 (13.3)	3156 (10.5)	2582 (13.0)
50–59	133 851 (30.8)	22 129 (34.3)	2824 (31.4)	21 720 (33.5)	74 408 (30.2)	6883 (23.0)	5887 (29.6)
60–69	127 193 (29.3)	17 591 (27.3)	2964 (33.0)	16 145 (24.9)	76 18 (31.0)	7696 (25.7)	6479 (32.6)
70–79	70 331 (16.2)	9466 (14.7)	1267 (14.1)	8450 (13.0)	40 975 (16.6)	6965 (23.2)	3208 (16.1)
≥ 80	37 590 (8.7)	3985 (6.2)	666 (7.4)	3973 (6.1)	21 986 (8.9)	5263 (17.6)	1717 (8.6)
Female (*N*, %)	221 638 (51.0)	28 319 (43.9)	4529 (50.4)	37 232 (57.5)	126 446 (51.3)	16 003 (53.4)	9109 (45.8)
Calendar year at entry (*N*, %)	—	—	—	—	—	—	—
2007	32 023 (7.4)	6951 (10.8)	48 (0.5)	8766 (13.5)	10 141 (4.1)	4811 (16.1)	1306 (6.6)
2008	31 014 (7.1)	7855 (12.2)	62 (0.7)	7663 (11.8)	10 317 (4.2)	4048 (13.5)	1069 (5.4)
2009	32 326 (7.4)	8038 (12.5)	64 (0.7)	7209 (11.1)	12 438 (5.0)	3432 (11.5)	1145 (5.8)
2010	32 748 (7.5)	6723 (10.4)	75 (0.8)	5664 (8.7)	16 914 (6.9)	2169 (7.2)	1203 (6.1)
2011	33 757 (7.8)	5778 (9.0)	121 (1.3)	5128 (7.9)	19 678 (8.0)	1799 (6.0)	1253 (6.3)
2012	34 709 (8.0)	5326 (8.3)	167 (1.9)	4563 (7.0)	21 672 (8.8)	1607 (5.4)	1374 (6.9)
2013	35 782 (8.2)	5101 (7.9)	195 (2.2)	4192 (6.5)	23 340 (9.5)	1596 (5.3)	1358 (6.8)
2014	34 457 (7.9)	4610 (7.2)	260 (2.9)	3890 (6.0)	22 688 (9.2)	1682 (5.6)	1327 (6.7)
2015	31 841 (7.3)	3562 (5.5)	686 (7.6)	3592 (5.5)	20 923 (8.5)	1682 (5.6)	1396 (7.0)
2016	32 908 (7.6)	3070 (4.8)	1452 (16.1)	3636 (5.6)	21 140 (8.6)	1779 (5.9)	1831 (9.2)
2017	33 229 (7.6)	2825 (4.4)	1682 (18.7)	3577 (5.5)	21 394 (8.7)	1820 (6.1)	1931 (9.7)
2018	35 216 (8.1)	2547 (4.0)	1997 (22.2)	3456 (5.3)	23 220 (9.4)	1761 (5.9)	2235 (11.2)
2019	34 496 (7.9)	2084 (3.2)	2183 (24.3)	3449 (5.3)	22 558 (9.2)	1777 (5.9)	2445 (12.3)
Follow‐up years (intention‐to‐treat censoring) (median, IQR)	6.2 [3.0, 9.6]	8.3 [5.0, 11.1]	2.8 [1.7, 4.2]	8.1 [4.2, 11.3]	5.7 [2.8, 8.6]	5.8 [1.3, 11.2]	5.0 [2.4, 8.7]
Baseline comorbidities (*N*, %)	—	—	—	—	—	—	—
Chronic obstructive pulmonary disease	9250 (2.1)	1086 (1.7)	121 (1.3)	455 (0.7)	5097 (2.1)	2364 (7.9)	127 (0.6)
Dyslipidaemia	62 834 (14.5)	16 438 (25.5)	2898 (32.2)	7396 (11.4)	31 358 (12.7)	2315 (7.7)	2429 (12.2)
Diabetes	68 398 (15.7)	31 422 (48.7)	3340 (37.1)	5283 (8.2)	23 589 (9.6)	2411 (8.0)	2353 (11.8)
Thyroid disorders	13 086 (3.0)	1300 (2.0)	234 (2.6)	4941 (7.6)	5522 (2.2)	782 (2.6)	307 (1.5)
Hypertension	242 391 (55.8)	32 548 (50.5)	5302 (59.0)	19 462 (30.0)	161 034 (65.3)	9802 (32.7)	14 243 (71.7)
Heart failure	3195 (0.7)	743 (1.2)	22 (0.2)	367 (0.6)	257 (0.1)	1593 (5.3)	213 (1.1)
Cerebrovascular diseases/stroke	24 035 (5.5)	6104 (9.5)	376 (4.2)	2649 (4.1)	13 097 (5.3)	1229 (4.1)	580 (2.9)
Ischaemic heart disease	15 863 (3.7)	3832 (5.9)	368 (4.1)	7373 (11.4)	2533 (1.0)	975 (3.3)	782 (3.9)
Peripheral vascular disease	1646 (0.4)	266 (0.4)	34 (0.4)	343 (0.5)	730 (0.3)	164 (0.5)	109 (0.5)
Arrhythmia and conduction disorders	12 902 (3.0)	1204 (1.9)	110 (1.2)	5258 (8.1)	4614 (1.9)	1319 (4.4)	397 (2.0)
Liver disease	10 622 (2.4)	1358 (2.1)	274 (3.0)	1872 (2.9)	4593 (1.9)	2221 (7.4)	304 (1.5)
Renal disease	8583 (2.0)	1060 (1.6)	200 (2.2)	1062 (1.6)	4853 (2.0)	1016 (3.4)	392 (2.0)
Schizophrenia and psychosis	6487 (1.5)	702 (1.1)	86 (1.0)	2627 (4.1)	2516 (1.0)	486 (1.6)	70 (0.4)
Bipolar disorder	866 (0.2)	81 (0.1)	17 (0.2)	380 (0.6)	301 (0.1)	78 (0.3)	9 (0.0)
Depression	9291 (2.1)	893 (1.4)	190 (2.1)	3450 (5.3)	3992 (1.6)	592 (2.0)	174 (0.9)
Anxiety disorder	5145 (1.2)	402 (0.6)	103 (1.1)	2230 (3.4)	2048 (0.8)	232 (0.8)	130 (0.7)
Smoking history	10 202 (2.3)	2318 (3.6)	214 (2.4)	1111 (1.7)	5581 (2.3)	773 (2.6)	205 (1.0)
Alcohol abuse disorder	2987 (0.7)	380 (0.6)	40 (0.4)	619 (1.0)	1320 (0.5)	537 (1.8)	91 (0.5)
Concurrent or previous use of drugs (90 days before index)	—	—	—	—	—	—	—
Antipsychotics	10 000 (2.3)	1032 (1.6)	113 (1.3)	4035 (6.2)	3588 (1.5)	1098 (3.7)	134 (0.7)
Antidepressants	14 870 (3.4)	1402 (2.2)	229 (2.5)	6271 (9.7)	5459 (2.2)	1106 (3.7)	403 (2.0)
Oral anticoagulants	2653 (0.6)	276 (0.4)	31 (0.3)	835 (1.3)	711 (0.3)	630 (2.1)	170 (0.9)
Antiplatelet drugs	46 547 (10.7)	10 957 (17.0)	1036 (11.5)	14 978 (23.1)	14 431 (5.9)	2708 (9.0)	2437 (12.3)
Insulins	7428 (1.7)	2784 (4.3)	282 (3.1)	954 (1.5)	2401 (1.0)	774 (2.6)	233 (1.2)
Non‐insulin antidiabetic drugs	65 046 (15.0)	30 946 (48.0)	3328 (37.0)	4797 (7.4)	21 168 (8.6)	2123 (7.1)	2684 (13.5)
Lipid‐regulating drugs	54 740 (12.6)	16 125 (25.0)	3378 (37.6)	9852 (15.2)	19 946 (8.1)	1757 (5.9)	3682 (18.5)
Thyroid and antithyroid drugs	11 522 (2.7)	1037 (1.6)	195 (2.2)	5352 (8.3)	3834 (1.6)	763 (2.5)	341 (1.7)
Anticholinergic drugs	71 627 (16.5)	9178 (14.2)	1059 (11.8)	13 733 (21.2)	39 031 (15.8)	6829 (22.8)	1797 (9.0)
Laboratory tests	—	—	—	—	—	—	—
Mean systolic blood pressure—12 months prior to index date (mmHg)	151.0 (17.0)	146.65 (14.54)	143.32 (15.41)	146.38 (15.43)	154.35 (16.98)	145.73 (17.33)	149.24 (19.12)
Mean diastolic blood pressure – 12 months prior to index date (mmHg)	85.3 (10.5)	83.65 (9.36)	82.21 (10.18)	83.42 (9.31)	86.87 (10.76)	81.67 (10.17)	84.34 (11.63)

Abbreviations: ACEI = Angiotensin‐Converting Enzyme Inhibitor, ARB = Angiotensin‐II Receptor Blocker, CCB = Calcium Channel Blocker, SD = Standard Deviation.

In the original study, we obtained estimated HRs suggesting there was no evidence of a difference in the hazard of all‐cause dementia across classes of antihypertensives when compared to the ACEI group. Under the HDPS approach, we identified approximately 1000 HDPS covariates from the specified data dimensions. A summary of IPTW including median, minimum, and maximum weights before and after trimming for each class comparison is shown in Table S2. In primary analyses incorporating the top 250 Bross‐ranked HDPS covariates, we did not observe major differences in the estimated HRs across comparisons. We found a shift to an association of lower hazard of all‐cause dementia with initiation of beta‐blockers compared to ACEI (HR: 0.90, 95% CI: 0.82–0.98) after inclusion of HDPS covariates in contrast with no evidence of an association using predefined covariates only (HR: 0.93, 95% CI: 0.86–1.02). The largest shift in point estimates (0.06) was observed in the diuretics and combination comparisons, respectively, after HDPS implementation, although this did not correspond with a change in conclusion.

The PS distributions before and after HDPS implementation are shown for each class comparison in Figures S1–S5. Noticeable shifts were observed in the ARB, beta‐blocker, and combination comparisons with ACEI. The change in distribution was most apparent in the beta‐blocker comparison where additional predictor variables for treatment initiation were captured by the HDPS process.

Absolute standardised differences (ASD) for pre‐defined covariates and the top 250 HDPS covariates were plotted out for each class comparison in Figures S6–S10. For any covariate, a difference of less than 10% between comparison groups was considered well‐balanced. When comparing models that were unweighted, weighted with pre‐defined covariates only, and weighted with pre‐defined covariates and top 250 HDPS covariates, the overall balance for all covariates was best achieved when weighting with the additional HDPS covariates. The unweighted ASDs were especially large for multiple pre‐defined and HDPS covariates in the beta‐blocker, CCB, and diuretic groups compared with the ACEI group, with many covariates exceeding ASDs of 20% and some exceeding 40%, but improved after weighting with HDPS covariates. In all models, it can also be observed that HDPS covariates ranked after the top 150 had relatively larger ASDs compared to pre‐defined and top 150 ranked HDPS covariates. Several of these HDPS covariates had non‐negligible differences (ASD > 10%) even after weighting with pre‐defined covariates only, while balance was improved when additionally weighting for top 250 HDPS covariates (Table 3).

**TABLE 3:** Estimated hazard ratios for association between different classes of antihypertensives and the risk of incident all‐cause dementia in original study and after application of high‐dimensional propensity scores.

Class	Number of individuals	Number of events	Unadjusted HR	IPTW, Adjusted HR (95% CI)	HDPS IPTW, Adjusted HR (95% CI)^a^
ACEI	64 470	2492	Ref.	Ref.	Ref.
ARB	8992	122	0.91 (0.76–1.09)	1.05 (0.79–1.39)	1.00 (0.77–1.31)
Beta‐blockers	64 785	1916	0.78 (0.74–0.83)	0.93 (0.86–1.02)	0.90 (0.82–0.98)
CCB	246 423	6939	1.00 (0.96–1.05)	0.96 (0.90–1.03)	0.96 (0.90–1.03)
Diuretics	29 963	1662	1.80 (1.69–1.91)	1.00 (0.91–1.10)	0.94 (0.85–1.03)
Combination	19 873	472	0.86 (0.78–0.95)	0.94 (0.83–1.07)	1.00 (0.87–1.16)

Abbreviations: ACEI = Angiotensin‐Converting Enzyme Inhibitor, ARB = Angiotensin‐II Receptor Blocker, CCB = Calcium Channel Blocker, CI = Confidence Interval, HDPS = High‐dimensional Propensity Score, HR = Hazard Ratio, IPTW = Inverse Probability of Treatment Weighting.

^a^ HDPS analysis adjusted for 250 covariates.

The top 20 Bross‐ranked HDPS covariates for each class comparison after covariate prioritisation are listed in Table S3 to describe the top covariates in terms of potential contribution to confounding bias. Laboratory measurements for estimated glomerular filtration rate (eGFR) were in the top 20 for each comparison, while uncomplicated hypertension (ICPC: K86), occlusion of cerebral arteries (ICD‐9: 434), and laboratory measurements for low‐density lipoprotein (LDL) cholesterol, urine albumin, and fasting blood sugar were in the top 20 for four out of five comparisons. The distributions of Bross bias values for the top 250 covariates for each class comparison are also shown in Figure S11.

The top three Bross‐ranked HDPS covariates that are also empirically IV‐like are listed in Table S4. These included several covariates related to diabetes diagnosis or treatment such as type 2 diabetes (ICPC: T90), sulphonylureas (BNF: 6.1.2.1), and other antidiabetic drugs (BNF: 6.1.2.3), commonly observed across class comparisons.

Plots comparing covariate‐outcome and covariate‐exposure associations for the top 250 covariates are shown in Figure S12 to identify potentially influential covariates with large associations. Visually assessed outliers included: (1) ARB vs. ACEI: eGFR laboratory tests, late effects of injuries to the nervous system (ICD‐9: 907) and fracture of the base of skull (ICD‐9: 801); (2) Beta‐blockers vs. ACEI: antithyroid drugs (BNF: 6.2.2) and antipsychotic depot injections (BNF: 4.2.2); (3) CCB vs. ACEI: protein‐calorie malnutrition (ICD‐9: 263); (4) Diuretics vs. ACEI: malignant neoplasm of the pancreas (ICD‐9: 157); (5) Combination vs. ACEI: intracranial haemorrhage (ICD‐9: 432).

In sensitivity analyses, we varied the number of HDPS covariates to 50, 100, 150, 200, and 500, and explored the impact of including the top 10 ranked HDPS covariates only and removing IV‐like covariates. From Table 4 and Table S5, the results remained relatively stable across the analyses for all class comparisons.

**TABLE 4:** Sensitivity analysis: varying the number of high‐dimensional propensity score variables included.

Class	IPTW, Adjusted HR (95% CI)	HDPS IPTW, Adjusted HR (95% CI) (50 covariates)	HDPS IPTW, Adjusted HR (95% CI) (100 covariates)	HDPS IPTW, Adjusted HR (95% CI) (150 covariates)	HDPS IPTW, Adjusted HR (95% CI) (200 covariates)	HDPS IPTW, Adjusted HR (95% CI) (250 covariates)	HDPS IPTW, Adjusted HR (95% CI) (500 covariates)
ACEI	Ref.	Ref.	Ref.	Ref.	Ref.	Ref.	Ref.
ARB	1.05 (0.79–1.39)	1.05 (0.81–1.36)	1.04 (0.80–1.35)	1.02 (0.79–1.33)	1.00 (0.77–1.31)	1.00 (0.77–1.31)	1.02 (0.78–1.34)
Beta‐blockers	0.93 (0.86–1.02)	0.89 (0.82–0.97)	0.88 (0.81–0.96)	0.88 (0.81–0.95)	0.89 (0.82–0.97)	0.90 (0.82–0.98)	0.90 (0.83–0.99)
CCB	0.96 (0.90–1.03)	0.97 (0.91–1.04)	0.97 (0.91–1.04)	0.98 (0.91–1.05)	0.96 (0.90–1.03)	0.96 (0.90–1.03)	0.97 (0.90–1.04)
Diuretics	1.00 (0.91–1.10)	0.92 (0.84–1.02)	0.92 (0.83–1.01)	0.93 (0.85–1.03)	0.93 (0.85–1.03)	0.94 (0.85–1.03)	0.94 (0.85–1.03)
Combination	0.94 (0.83–1.07)	1.00 (0.87–1.14)	0.99 (0.86–1.14)	1.00 (0.86–1.15)	1.00 (0.87–1.16)	1.00 (0.87–1.16)	0.97 (0.84–1.12)

Abbreviations: ACEI = Angiotensin‐Converting Enzyme Inhibitor, ARB = Angiotensin‐II Receptor Blocker, CCB = Calcium Channel Blocker, CI = Confidence Interval, HDPS = High‐dimensional Propensity Score, HR = Hazard Ratio, IPTW = Inverse Probability of Treatment Weighting.

## 5 Discussion

We implemented HDPS methods and principles in this study to electronic health data in HK, taking a study including relatively older people where residual confounding was likely between comparison groups. We also developed an R package to facilitate conducting HDPS analysis in versatile settings for future studies.

This study highlights the potential of the HDPS implementation process to identify covariates in a database that may be overlooked or neglected by investigators when choosing variables to be included in PS or model adjustment. Diagnostic codes such as protein‐calorie malnutrition and skull fractures are potential indicators of frailty present in older adults and would not usually be identified in such a study using conventional PS methods. Additionally, laboratory measurements such as eGFR and albumin tests (regardless of test results) were among the top ranked HDPS covariates, which have been noted as potential biomarkers of frailty [26, 27]. This highlights the potential of HDPS, in a specific database, to identify potentially important covariates which may be proxies for concepts that can lead to unmeasured confounding. If similar covariates are consistently identified across different studies, this could lead to the more routine consideration of these covariates as proxies for frailty in the database under investigation. However, these covariates remain as proxies instead of direct measures of frailty and the degree to which these proxies capture the full spectrum of frailty is unknown, meaning that residual confounding from this factor can still persist.

Overall, we observed minor changes in the effect estimates after the inclusion of HDPS covariates compared with PS models with only pre‐defined investigator variables. However, we observed that the association shifted from no evidence to moderate evidence of a lower hazard of all‐cause dementia comparing beta‐blockers with ACEI. This association was not detected using investigator pre‐specified covariates only, although this shift may not truly reflect a change in the causal relationship and the association should be further verified. There has been evidence of beta‐blocker use having a lower hazard of dementia compared with ACEI in another large study in the Netherlands [20]. The PS distribution diagnostic also showed a change in distribution in this comparison that could be attributed to additional covariates including blood cell counts from laboratory measurements selected from the HDPS process, which were among the top 20 HDPS covariates with confounding implications (Table S1). Although this pattern alone does not indicate an improved confounding control, the inclusion of laboratory measurement data is also useful in enriching the data and providing indicators of disease severity in addition to diagnostic codes.

Interestingly, highly Bross‐ranked HDPS covariates which were empirically IV‐like included covariates related to type 2 diabetes for all comparison groups. ACEI, along with ARBs, are preferred antihypertensive classes for hypertension in patients with type 2 diabetes [28, 29], which is a possible link with why this was observed. However, diabetes is recognised as a midlife risk factor for dementia [30] and diabetes‐related covariates should theoretically be confounders rather than the hypothesised IVs. Methods and thresholds used to explore empirically IV‐like variables in HDPS should be further explored and evaluated.

One of the common concerns of HDPS is that variables such as colliders (variables that are influenced by two or more other variables) and IVs may be included in the PS, which introduces the potential for M‐bias and Z‐bias, respectively. These biases have been shown to be small in magnitude for the most commonly encountered settings in pharmacoepidemiology studies [31, 32]. In this study, results remained consistent after removal of HDPS covariates that behaved like IVs in sensitivity analyses. Furthermore, it is considered that the true threat to study validity in non‐randomised pharmacoepidemiology is unmeasured confounding, which the HDPS method attempts to mitigate, and that the benefit of including a large number of covariates through data‐driven selection for confounding adjustment may often outweigh the harm of including inappropriate variables [33].

Another criticism of the HDPS approach is that it appears to be a black box with low transparency with regards to details in the implementation process. In this study, the specifications of each step of the HDPS algorithm during the implementation process were clearly documented and reported following recent guidance on the reporting considerations for HDPS [10, 11]. Various diagnostic tools and sensitivity analyses were also used to explore the properties and influence of HDPS covariates on the generated results. Furthermore, an R package was developed with the aim of producing reproducible results and improving the ease of uptake of this method.

In this study, we implemented HDPS principles to routinely collected clinical data from hospital settings in HK. Understanding healthcare databases, the associated healthcare system, and the level of data capture is important as limitations of each database can also translate into limitations of the HDPS. Such limitations in the HK data include possible missing data from laboratory measurements and the absence of data from private healthcare services. Validation of dementia diagnoses has also not been performed in the HK data, but other diagnoses such as stroke and myocardial infarction with high positive predictive values suggest the validity of the data [16, 17]. However, any misclassification of the outcome between antihypertensive groups is expected to be non‐differential and bias the estimates towards the null. Although the HDPS can extract all variables with potential for confounding available in a specific database, it does not solve issues involving missing data and lack of information in the database.

There can also be challenges when implementing HDPS to other healthcare systems or databases with varying data structures or diverse coding systems. As the HDPS was originally developed for administrative claims data, extensions were required to implement HDPS methods with the richer clinical details in electronic health records [21]. In this study, laboratory measurements were included as a data dimension from the secondary data available in HK to provide proxies of disease severity alongside diagnosis and prescription data. HDPS has also been adapted for implementation in a national health survey setting [34], which required restructuring of the dataset and the generation of a dataset‐specific coding system to resemble claims or electronic health record data. Regarding our R package, its usage is generalisable with any database, assuming that such restructuring of data and organisation of coding systems have been conducted beforehand to fit the data format in the documented simulated data examples provided with the package.

Developments to HDPS methods have been made since it was originally introduced. This study mainly followed the original algorithm with the exception of not applying the prevalence filter. Machine learning methods to improve the confounder selection process have also been suggested to be incorporated with HDPS [35]. Future work could explore the application of these methods in the context of HK data, with the potential to leverage untapped data, such as unstructured free‐text data and continuous variables.

Overall, this study demonstrated the implementation of HDPS in HK electronic health records and showed the potential advantage of this method to contribute to improved confounding adjustment and epidemiological knowledge. We hope this work provides a foundation for future studies in HK and other similar settings with healthcare databases that include both primary and secondary care data.

## Author Contributions

E.C.L.C., A.Y.S.W., and J.T. were responsible for the study concept and developed the study approach. E.C.L.C. reviewed the literature, conducted the statistical analysis, and wrote the first draft of the manuscript. M.F. was responsible for the software package development. M.F., C.S.L.C., A.Y.S.W., and J.T. provided critical input to the study design, analyses, and discussion. All authors contributed to the interpretation of the analyses, critically reviewed and revised the intellectual content, and were involved in the final approval of the manuscript.

## Funding

This work was supported by the Hong Kong Research Grants Council General Research Fund [grant number 17113720]. This study is also supported by the Laboratory of Data Discovery for Health (D24H) funded by AIR@InnoHK administered by the Innovation and Technology Commission, the Government of the Hong Kong Special Administrative Region, China. John Tazare was funded by the Wellcome Trust [grant 224 485/Z/21/Z]. The study sponsors were not involved in study design; in the collection, analysis, and interpretation of data; in the writing of the report; and in the decision to submit the paper for publication.

## Ethics Statement

This study was approved by the Institutional Review Board of the University of Hong Kong/Hospital Authority West Cluster (Reference number: UW 20–053).

## Conflicts of Interest

C.S.L.C. has received grants from the Food and Health Bureau of the Hong Kong Government, Hong Kong Research Grant Council, Hong Kong Innovation and Technology Commission, Pfizer, IQVIA, and Amgen; personal fees from Primevigilance Ltd.; outside the submitted work.

## Supporting information


**Figure S1:** Estimated propensity scores before and after additional inclusion of 250 HDPS covariates (Angiotensin‐II receptor blockers versus angiotensin‐converting enzyme inhibitors).
**Figure S2:** Estimated propensity scores before and after additional inclusion of 250 HDPS covariates (Beta‐blockers versus angiotensin‐converting enzyme inhibitors).
**Figure S3:** Estimated propensity scores before and after additional inclusion of 250 HDPS covariates (Calcium channel blockers versus angiotensin‐converting enzyme inhibitors).
**Figure S4:** Estimated propensity scores before and after additional inclusion of 250 HDPS covariates (Diuretics versus angiotensin‐converting enzyme inhibitors).
**Figure S5:** Estimated propensity scores before and after additional inclusion of 250 HDPS covariates (Combination versus angiotensin‐converting enzyme inhibitors).
**Figure S6:** Absolute standardised differences compared between propensity score models including unweighted, pre‐defined variables only, and pre‐defined variables with additional high‐dimensional propensity score covariates (Angiotensin‐II receptor blockers versus angiotensin‐converting enzyme inhibitors).
**Figure S7:** Absolute standardised differences compared between propensity score models including unweighted, pre‐defined variables only, and pre‐defined variables with additional high‐dimensional propensity score covariates (Beta‐blockers versus angiotensin‐converting enzyme inhibitors).
**Figure S8:** Absolute standardised differences compared between propensity score models including unweighted, pre‐defined variables only, and pre‐defined variables with additional high‐dimensional propensity score covariates (Calcium channel blockers versus angiotensin‐converting enzyme inhibitors).
**Figure S9:** Absolute standardised differences compared between propensity score models including unweighted, pre‐defined variables only, and pre‐defined variables with additional high‐dimensional propensity score covariates (Diuretics versus angiotensin‐converting enzyme inhibitors).
**Figure S10:** Absolute standardised differences compared between propensity score models including unweighted, pre‐defined variables only, and pre‐defined variables with additional high‐dimensional propensity score covariates (Combination versus angiotensin‐converting enzyme inhibitors).
**Figure S11:** Distribution of absolute log Bross bias values for the top 250 high‐dimensional propensity score covariates in each antihypertensive class comparison.
**Figure S12:** Plots of covariate‐outcome versus covariate‐exposure associations for top 250 high‐dimensional propensity score covariates in each antihypertensive class comparison.
**Table S1:** Timed comparison of executing a combination of candidate feature identification, recurrence assessment, and covariate prioritisation functions between our package (cainefm/hdps) and existing R packages (technOslerphile/autoCovariateSelection and lendle/hdps).
**Table S2:** Summary of inverse probability of treatment weighting with additional high‐dimensional propensity score covariates before and after weight trimming for each antihypertensive class comparison.
**Table S3:** List of top 20 high‐dimensional propensity score covariates for each antihypertensive class comparison after Bross formula‐based covariate prioritisation.
**Table S4:** List of top 3 Bross‐ranked high‐dimensional propensity score covariates for each antihypertensive class comparison that are empirically strongly related with exposure but not the outcome.
**Table S5:** Sensitivity analysis: Exploring potentially influential high‐dimensional propensity score covariates.

**Data S1:** pds70326‐sup‐0002‐Supinfo2.gz.

## Data Availability

Data will not be made available to others as the data custodians have not given permission.
