# Supplementary material for: High‐Dimensional Propensity Scores for Mitigating Confounding: Implementation Using Primary and Secondary Care Data in Hong Kong
Source: Pharmacoepidemiol Drug Saf. 2026 Jan 25;35(2):e70326. doi: 10.1002/pds.70326 (PMC12833473; doi:10.1002/pds.70326)
Supplement: Supplementary file 1 — Figure S1: Estimated propensity scores before and after additional inclusion of 250 HDPS covariates (Angiotensin‐II receptor blockers versus angiotensin‐converting enzyme inhibitors). Figure S2: Estimated propensity scores before and after additional inclusion of 250 HDPS covariates (Beta‐blockers versus angiotensin‐converting enzyme inhibitors). Figure S3: Estimated propensity scores before and after additional inclusion of 250 HDPS covariates (Calcium channel blockers versus angiotensin‐converting enzyme inhibitors). Figure S4: Estimated propensity scores before and after additional inclusion of 250 HDPS covariates (Diuretics versus angiotensin‐converting enzyme inhibitors). Figure S5: Estimated propensity scores before and after additional inclusion of 250 HDPS covariates (Combination versus angiotensin‐converting enzyme inhibitors). Figure S6: Absolute standardised differences compared between propensity score models including unweighted, pre‐defined variables only, and pre‐defined variables with additional high‐dimensional propensity score covariates (Angiotensin‐II receptor blockers versus angiotensin‐converting enzyme inhibitors). Figure S7: Absolute standardised differences compared between propensity score models including unweighted, pre‐defined variables only, and pre‐defined variables with additional high‐dimensional propensity score covariates (Beta‐blockers versus angiotensin‐converting enzyme inhibitors). Figure S8: Absolute standardised differences compared between propensity score models including unweighted, pre‐defined variables only, and pre‐defined variables with additional high‐dimensional propensity score covariates (Calcium channel blockers versus angiotensin‐converting enzyme inhibitors). Figure S9: Absolute standardised differences compared between propensity score models including unweighted, pre‐defined variables only, and pre‐defined variables with additional high‐dimensional propensity score covariates (Diuretics versus angiotensin‐conv [file PDS-35-e70326-s001.docx]

Supplementary Figure 1. Estimated propensity scores before and after additional inclusion of 250 HDPS covariates (Angiotensin-II receptor blockers versus angiotensin-converting enzyme inhibitors)


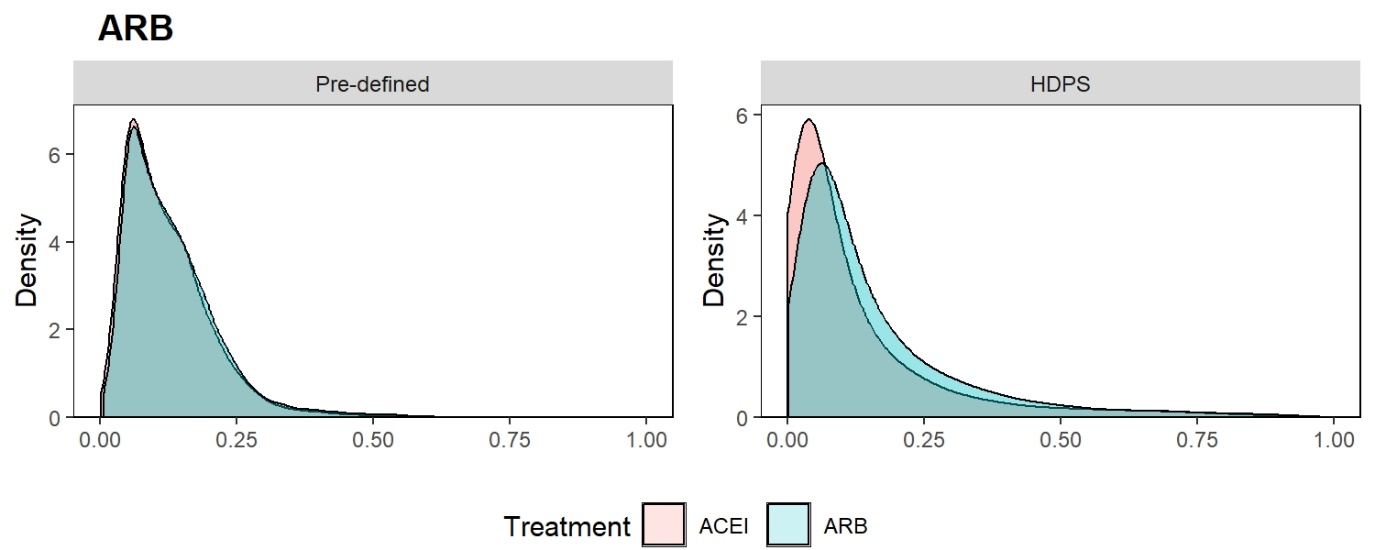


Abbreviations: ARB = Angiotensin-II Receptor Blocker, ACEI = Angiotensin-Converting Enzyme Inhibitor, HDPS = High-dimensional Propensity Score

Supplementary Figure 2. Estimated propensity scores before and after additional inclusion of 250 HDPS covariates (Beta-blockers versus angiotensin-converting enzyme inhibitors)


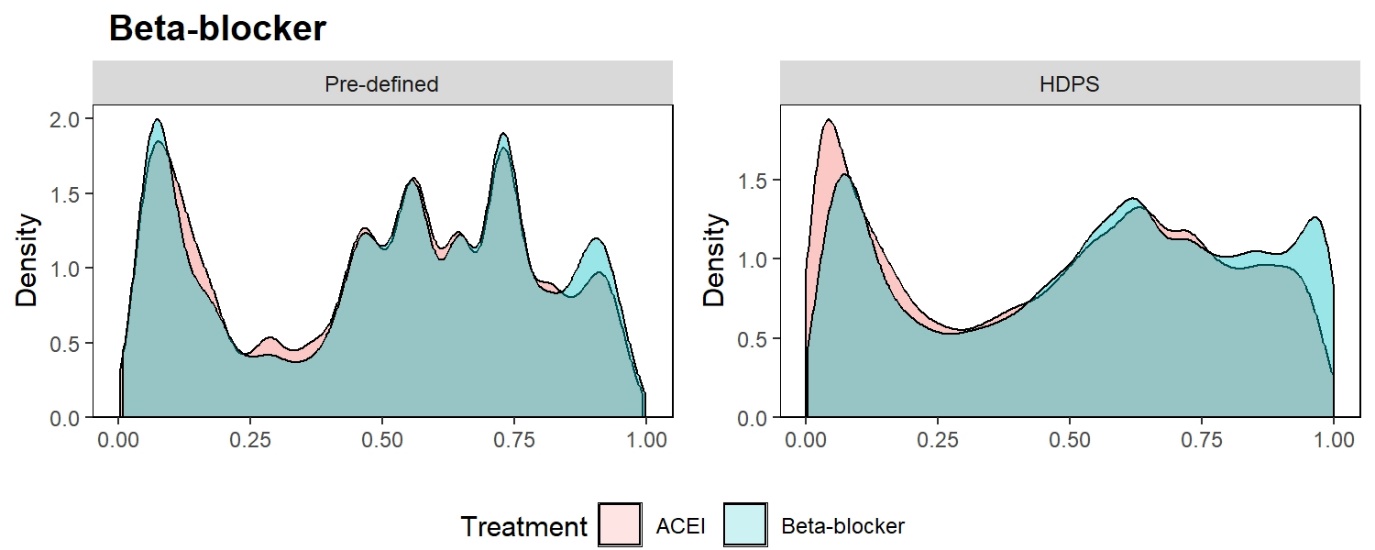


Abbreviations: ACEI = Angiotensin-Converting Enzyme Inhibitor, HDPS = High-dimensional Propensity Score

Supplementary Figure 3. Estimated propensity scores before and after additional inclusion of 250 HDPS covariates (Calcium channel blockers versus angiotensin-converting enzyme inhibitors)


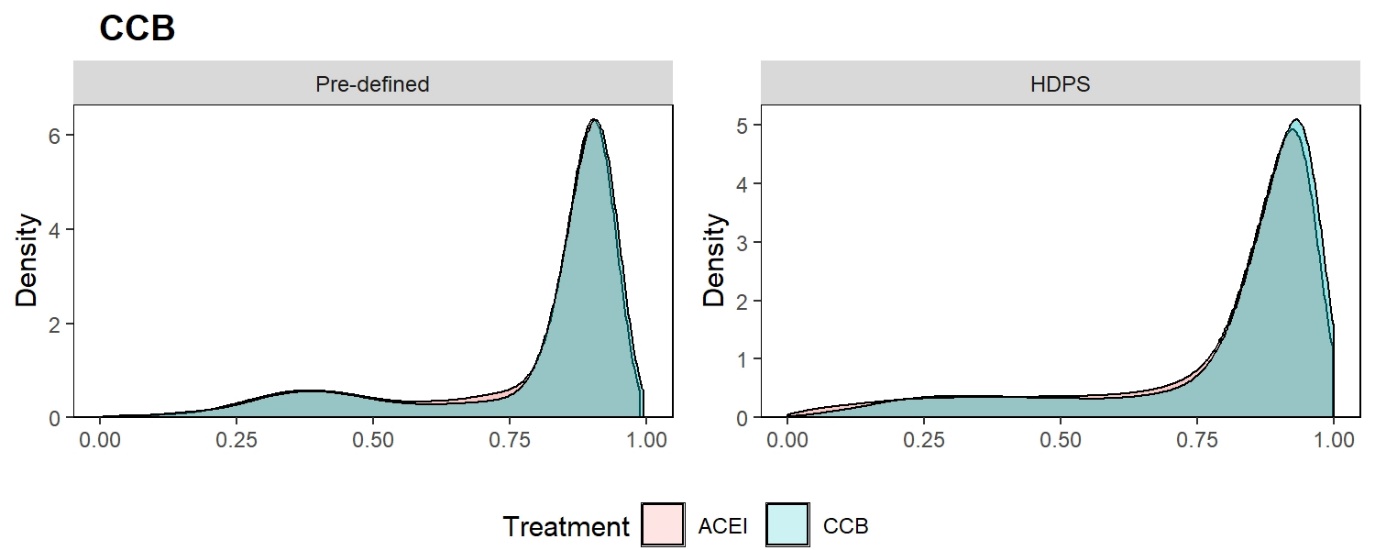


Abbreviations: CCB = Calcium channel blockers, ACEI = Angiotensin-Converting Enzyme Inhibitor, HDPS = High-dimensional Propensity Score

Supplementary Figure 4. Estimated propensity scores before and after additional inclusion of 250 HDPS covariates (Diuretics versus angiotensin-converting enzyme inhibitors)


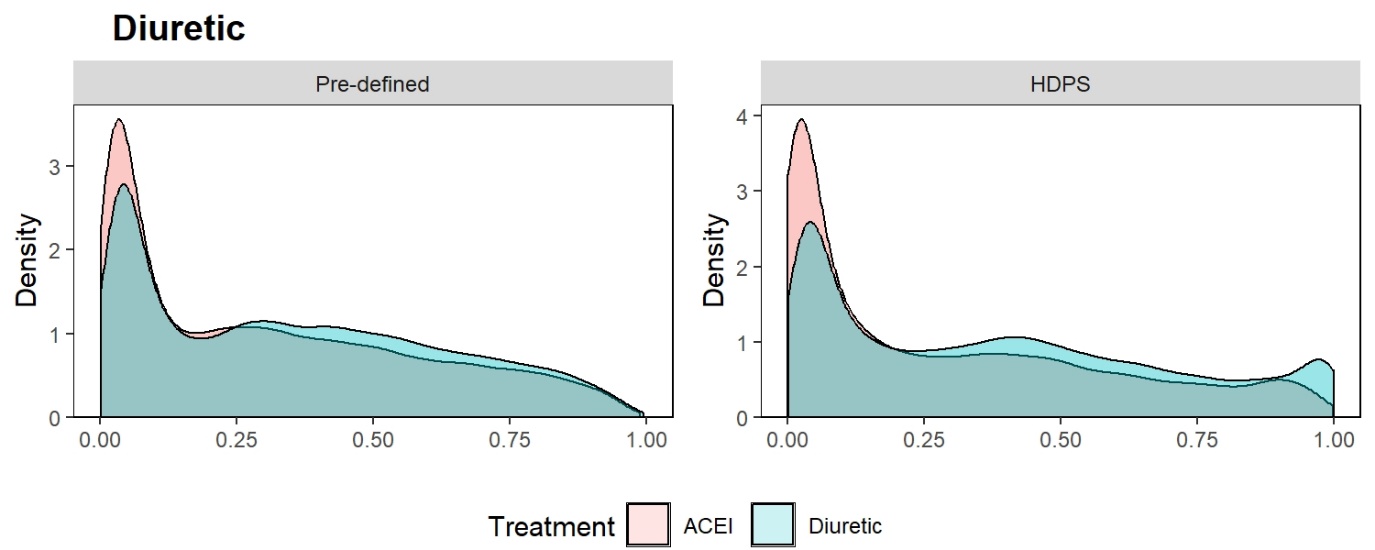


Abbreviations: ACEI = Angiotensin-Converting Enzyme Inhibitor, HDPS = High-dimensional Propensity Score

Supplementary Figure 5. Estimated propensity scores before and after additional inclusion of 250 HDPS covariates (Combination versus angiotensin-converting enzyme inhibitors)


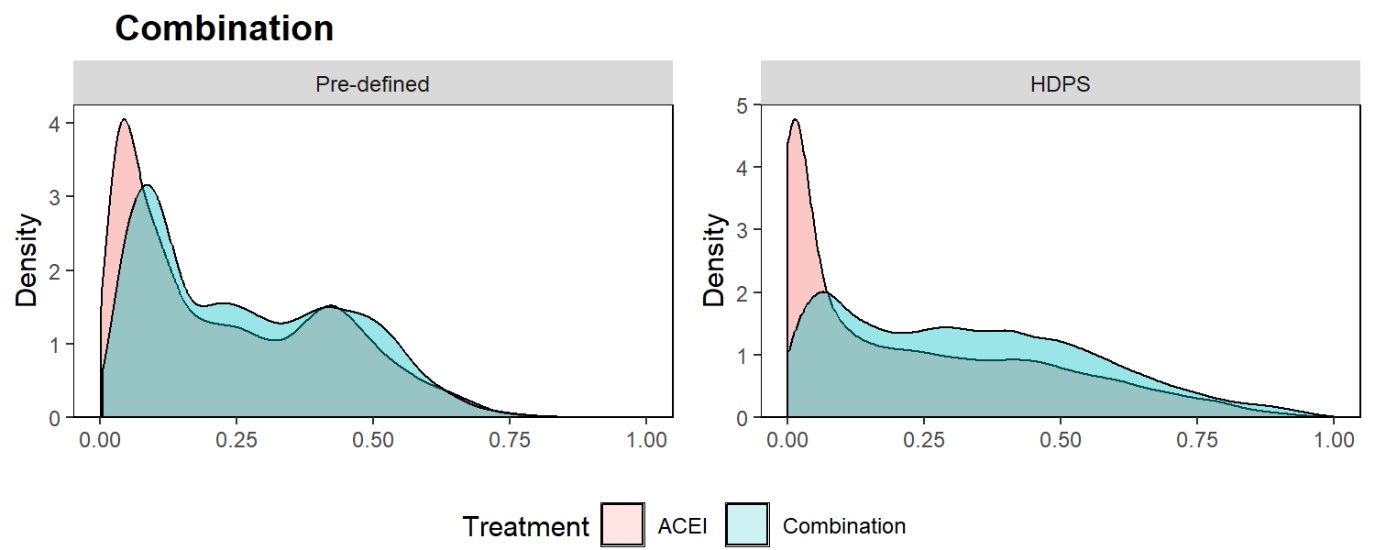


Abbreviations: ACEI = Angiotensin-Converting Enzyme Inhibitor, HDPS = High-dimensional Propensity Score

Supplementary Figure 6. Absolute standardized differences compared between propensity score models including unweighted, pre-defined variables only, and pre-defined variables with additional high-dimensional propensity score covariates

(Angiotensin-II receptor blockers versus angiotensin-converting enzyme inhibitors)


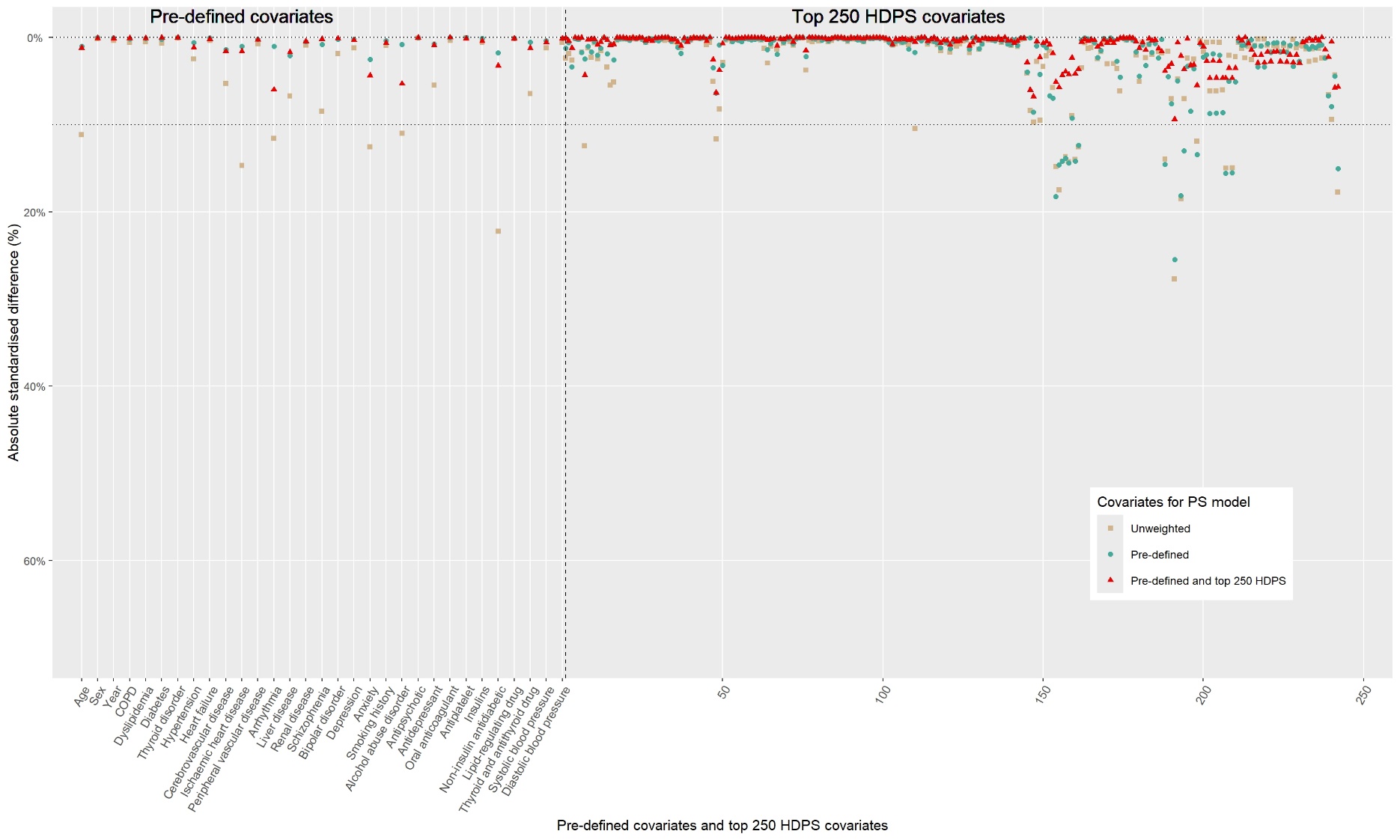


Abbreviations: HDPS = High-dimensional Propensity Score, PS = Propensity Score

Supplementary Figure 7. Absolute standardized differences compared between propensity score models including unweighted, pre-defined variables only, and pre-defined variables with additional high-dimensional propensity score covariates

(Beta-blockers versus angiotensin-converting enzyme inhibitors)


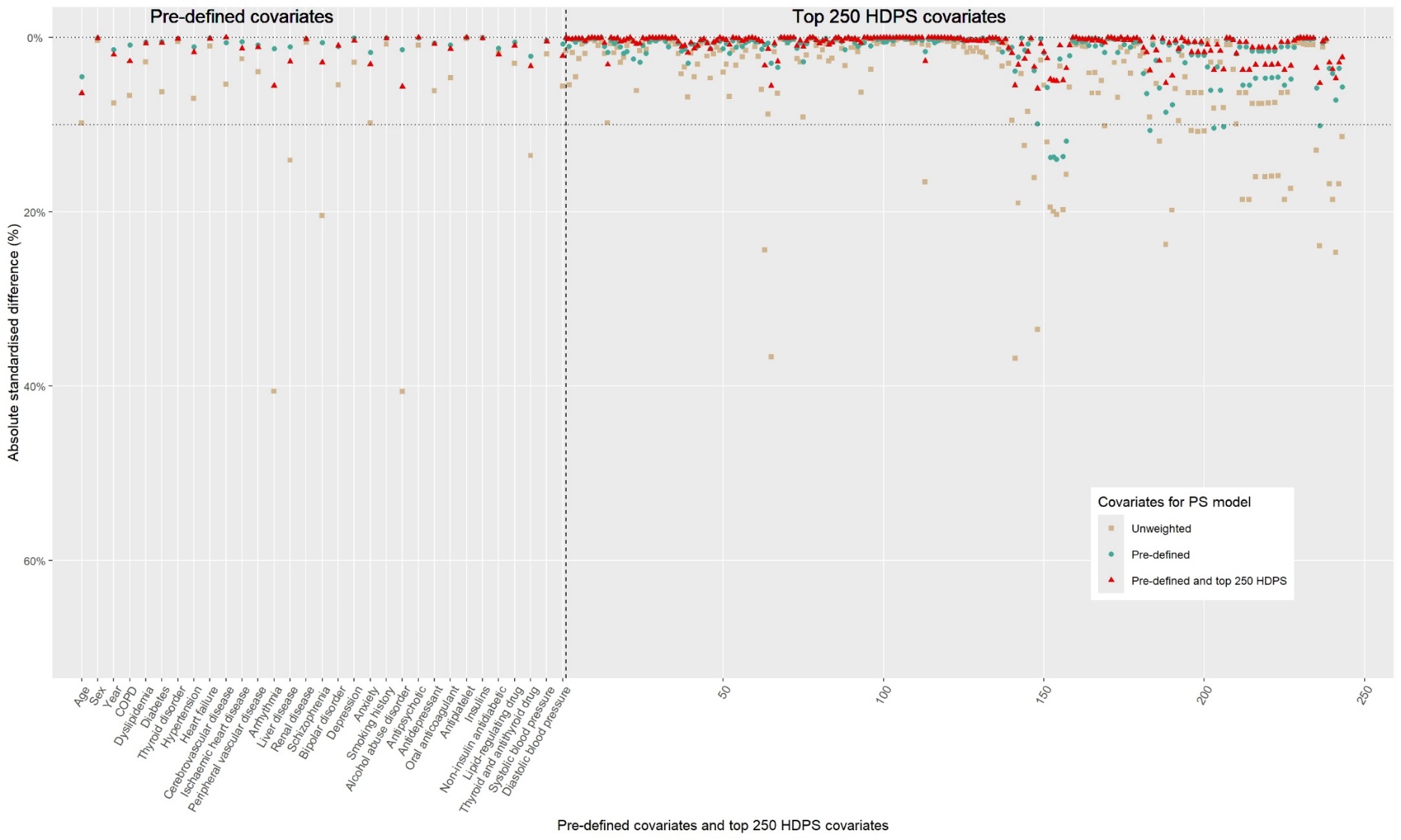


Abbreviations: HDPS = High-dimensional Propensity Score, PS = Propensity Score

Supplementary Figure 8. Absolute standardized differences compared between propensity score models including unweighted, pre-defined variables only, and pre-defined variables with additional high-dimensional propensity score covariates

(Calcium channel blockers versus angiotensin-converting enzyme inhibitors)


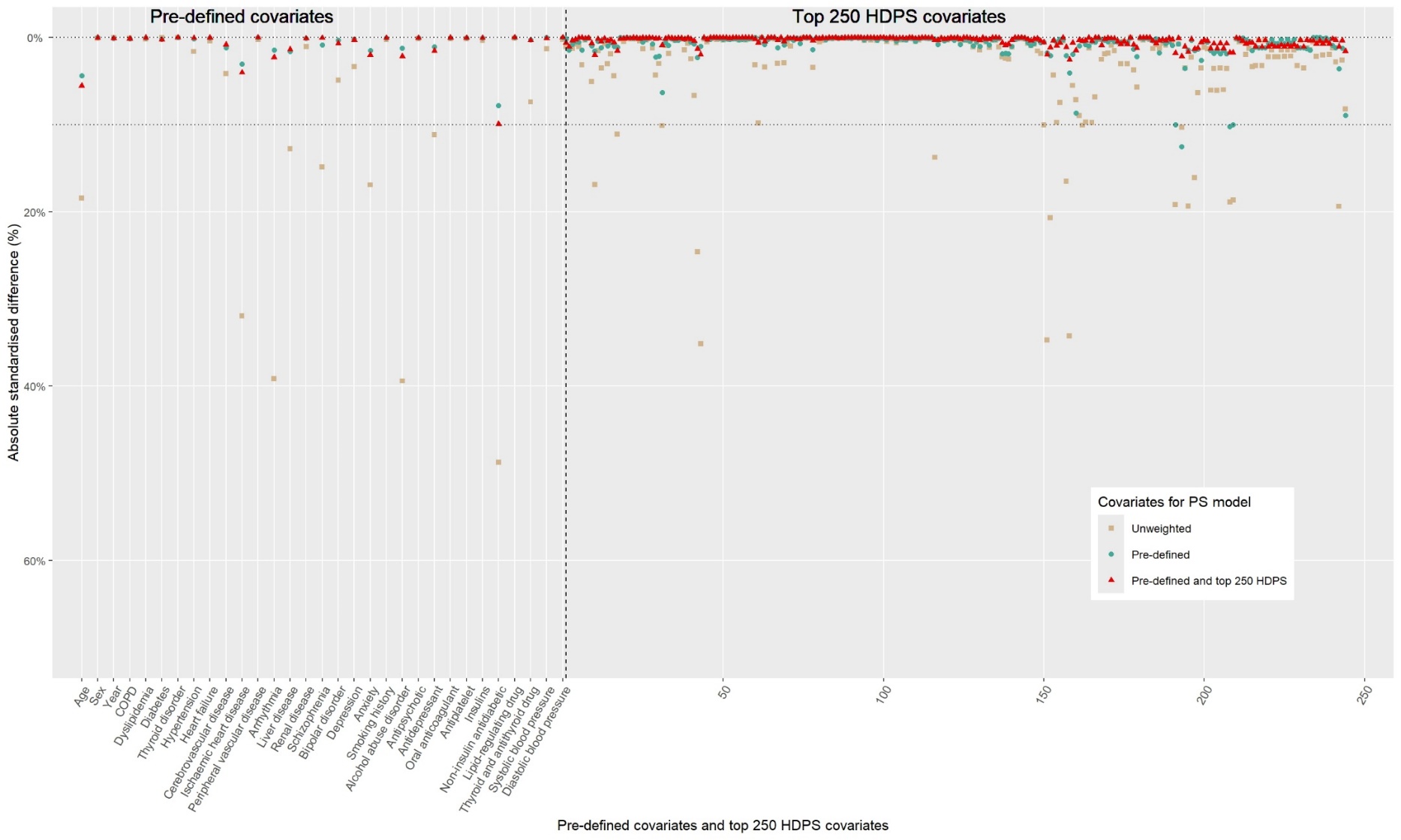


Abbreviations: HDPS = High-dimensional Propensity Score, PS = Propensity Score

Supplementary Figure 9. Absolute standardized differences compared between propensity score models including unweighted, pre-defined variables only, and pre-defined variables with additional high-dimensional propensity score covariates

(Diuretics versus angiotensin-converting enzyme inhibitors)
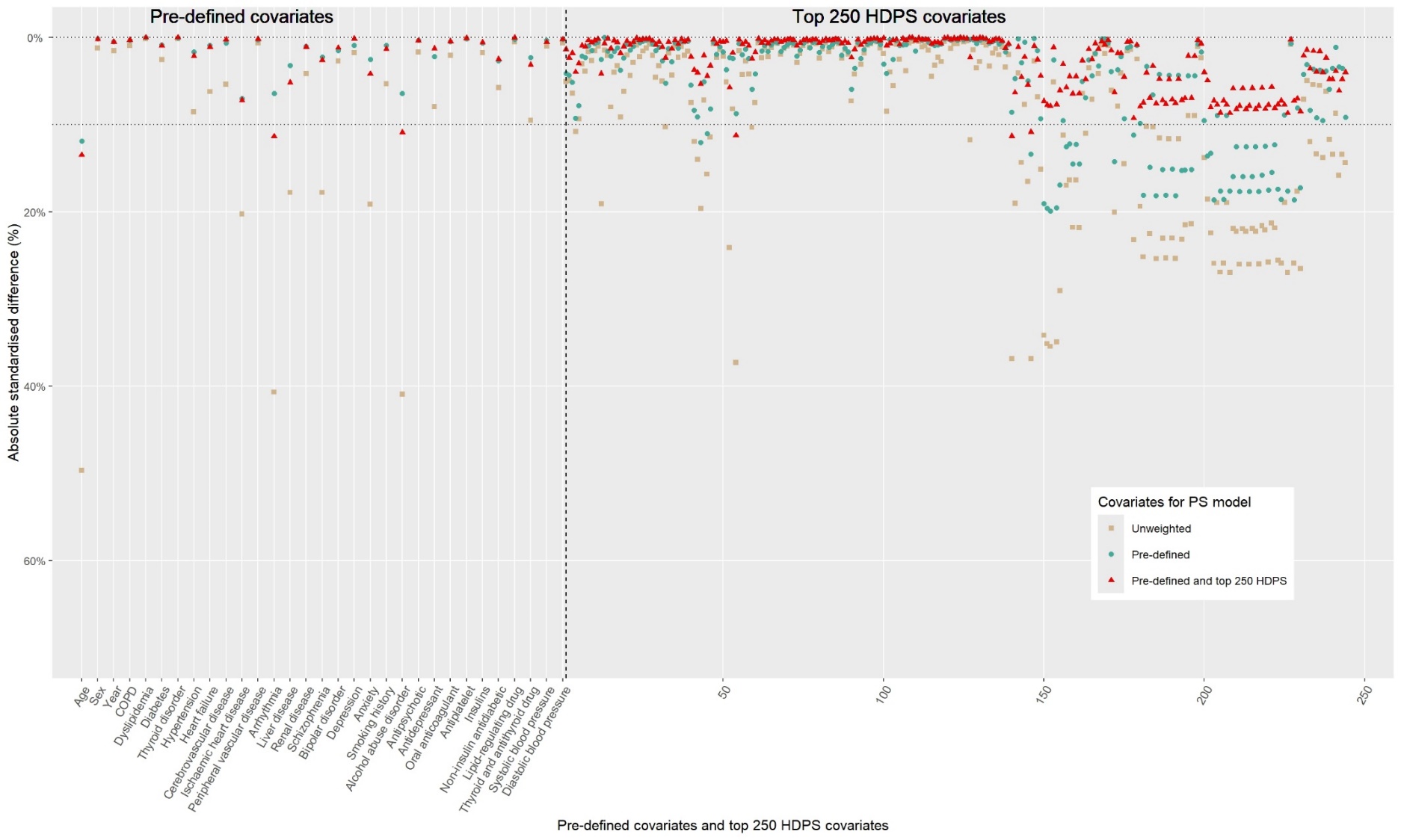


Abbreviations: HDPS = High-dimensional Propensity Score, PS = Propensity Score

Supplementary Figure 10. Absolute standardized differences compared between propensity score models including unweighted, pre-defined variables only, and pre-defined variables with additional high-dimensional propensity score covariates

(Combination versus angiotensin-converting enzyme inhibitors)
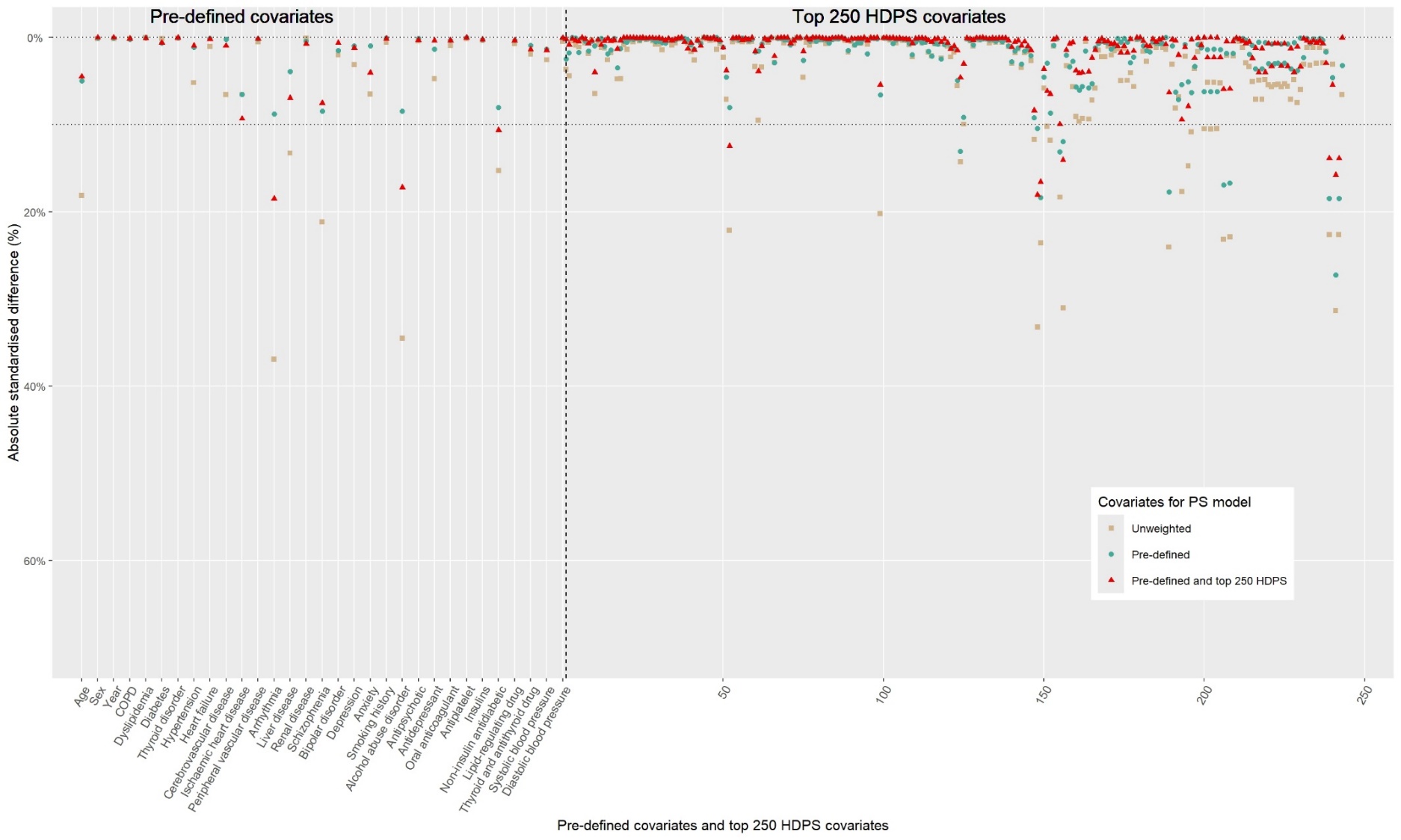


Abbreviations: HDPS = High-dimensional Propensity Score, PS = Propensity Score

Supplementary Figure 11. Distribution of absolute log Bross bias values for the top 250 high-dimensional propensity score covariates in each antihypertensive class comparison
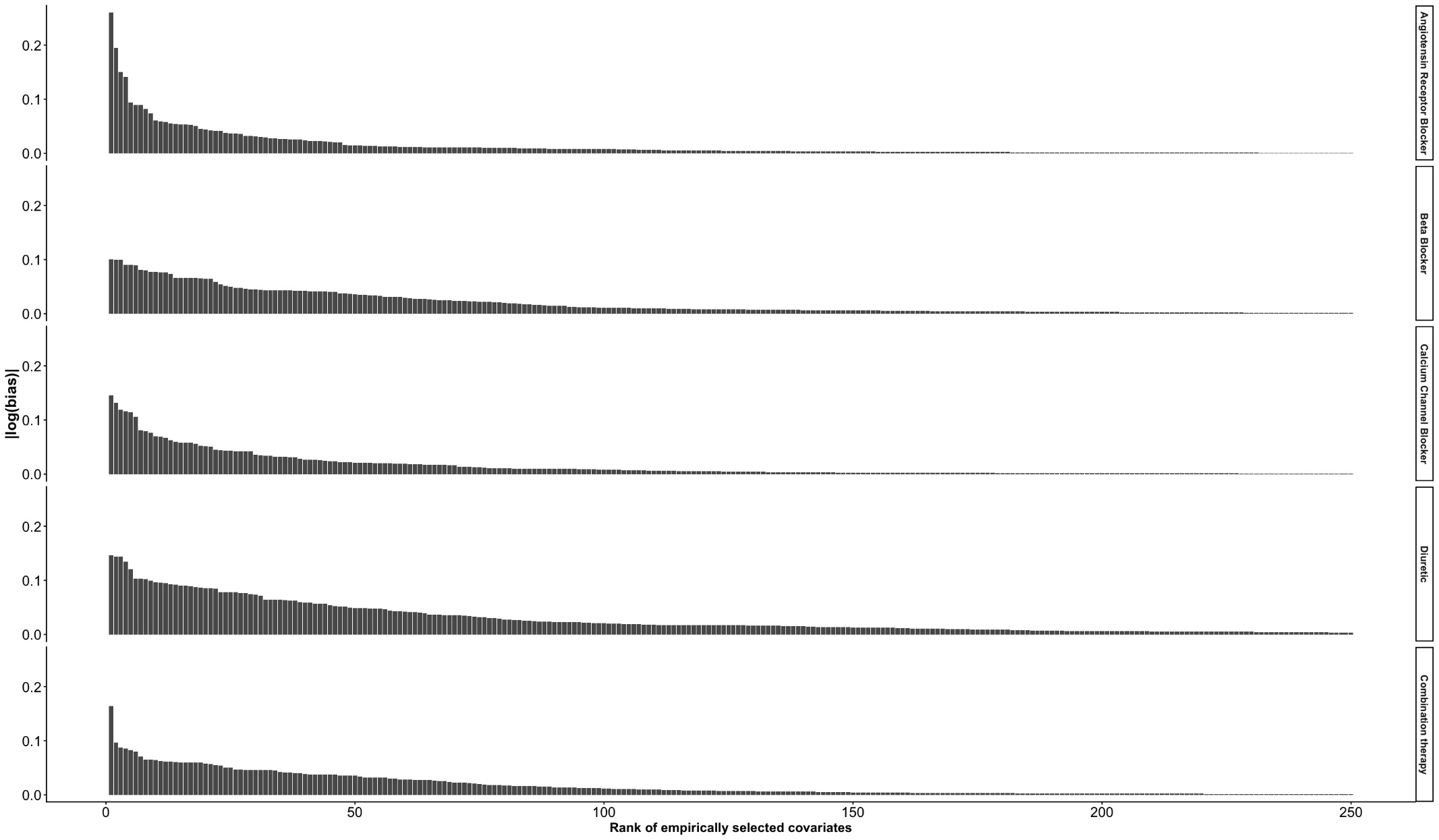


Supplementary Figure 12. Plots of covariate-outcome versus covariate-exposure associations for top 250 high-dimensional propensity score covariates in each antihypertensive class comparison


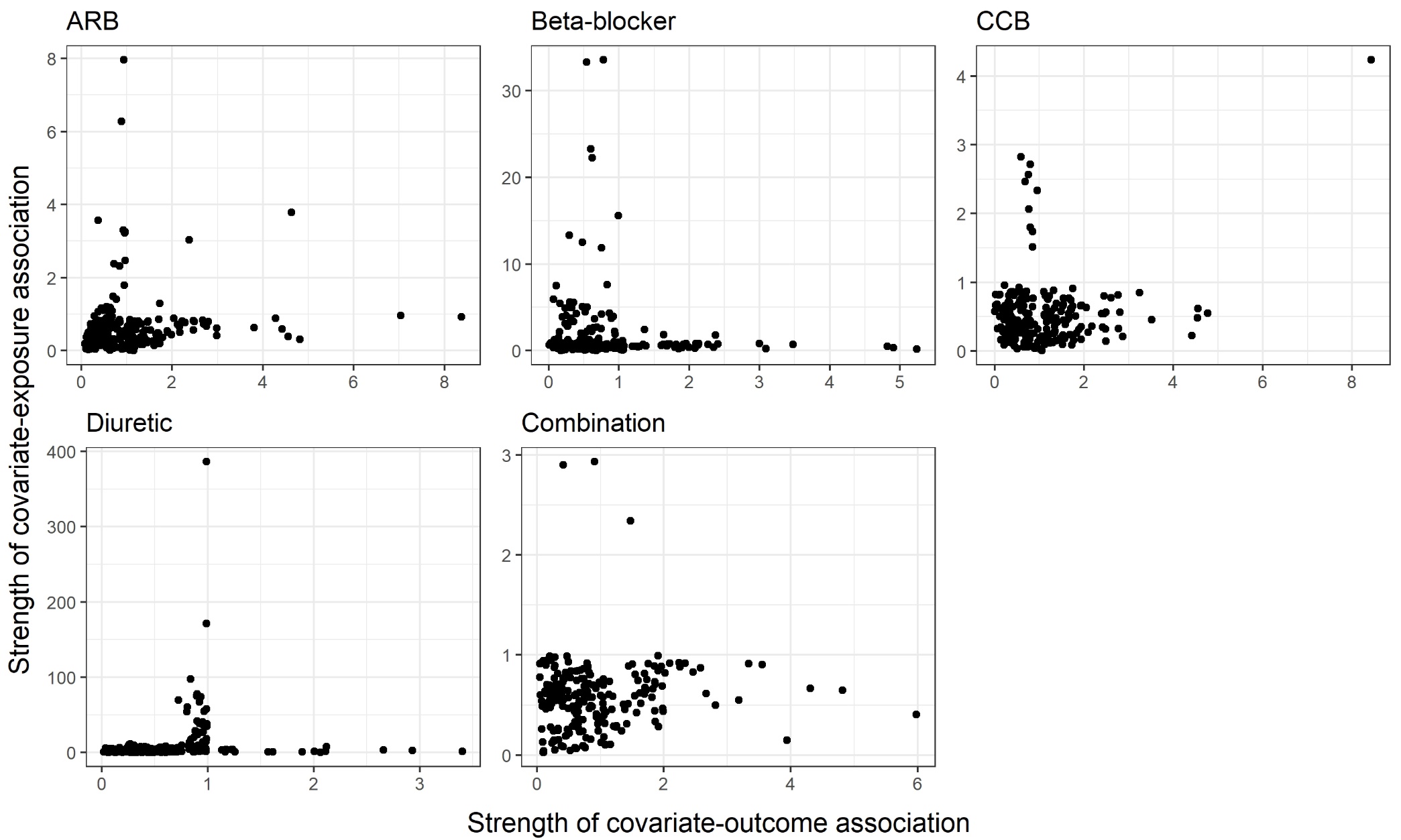


Supplementary Table 1. Timed comparison of executing a combination of candidate feature identification, recurrence assessment, and covariate prioritisation functions between our package (cainefm/hdps) and existing R packages (technOslerphile/autoCovariateSelection and lendle/hdps)

| **Dataset size by number of individuals** | **cainefm/hdps** | | **technOslerphile/autoCovariateSelection** | | **lendle/hdps** | |
| --- | --- | --- | --- | --- | --- | --- |
|  | **Time taken (s)** | **RAM used (MB)** | **Time taken (s)** | **RAM used (MB)** | **Time taken (s)** | **RAM used (MB)** |
| 500 | 1.2 | 45 | 2.8 | 78 | 1.8 | 62 |
| 1,000 | 2.5 | 89 | 5.2 | 156 | 3.8 | 124 |
| 3,000 | 8.3 | 267 | 18.7 | 468 | 12.5 | 372 |
| 10,000 | 28.5 | 890 | 65.2 | 1,560 | 42.8 | 1,240 |

Abbreviations: s = seconds, RAM = Random Access Memory, MB = megabytes

Supplementary Table 2. Summary of inverse probability of treatment weighting with additional high-dimensional propensity score covariates before and after weight trimming for each antihypertensive class comparison

| **Class comparison vs ACEI** | **Untrimmed weights** | | | **Weights trimmed at the 99^th^ percentile** | | |
| --- | --- | --- | --- | --- | --- | --- |
|  | **Median** | **Minimum** | **Maximum** | **Median** | **Minimum** | **Maximum** |
| ARB | 0.89 | 0.13 | 187.4 | 0.89 | 0.16 | 3.27 |
| Beta-blockers | 0.66 | 0.50 | 379.4 | 0.66 | 0.50 | 6.74 |
| CCB | 0.85 | 0.21 | 852.5 | 0.85 | 0.22 | 4.80 |
| Diuretics | 0.71 | 0.32 | 1326.3 | 0.71 | 0.32 | 6.80 |
| Combination | 0.79 | 0.24 | 2256.29 | 0.79 | 0.27 | 4.14 |

Abbreviations: ACEI = Angiotensin-Converting Enzyme Inhibitor, ARB = Angiotensin-II Receptor Blocker, CCB = Calcium Channel Blocker

Supplementary Table 3. List of top 20 high-dimensional propensity score covariates for each antihypertensive class comparison after Bross formula-based covariate prioritisation

|  | **Comparison vs ACEI** | | | | | | | | | |
| --- | --- | --- | --- | --- | --- | --- | --- | --- | --- | --- |
| **Top 20 HDPS covariates** | **ARB** | | **Beta-blockers** | | **CCB** | | **Diuretics** | | **Combination** | |
|  | **Covariate** | **R** | **Covariate** | **R** | **Covariate** | **R** | **Covariate** | **R** | **Covariate** | **R** |
| 1 | L – eGFR (MDRD) | 1 | P – BNF 6.1.2.1: Sulphonylureas | 1 | P – BNF 2.9: Antiplatelet drugs | 1 | L – Cholesterol, HDL | 1 | D – ICPC K86: Hypertension uncomplicated | 1 |
| 2 | L – eGFR (CKD-EPI) | 1 | L – BP (Systolic) | 1 | P – BNF 6.1.2.1: Sulphonylureas | 1 | L – Triglycerides | 1 | D – ICD-9 434: Occlusion of cerebral arteries | 1 |
| 3 | L – Fasting Blood Sugar | 1 | L – BP (Diastolic) | 1 | L – Sodium | 3 | L – Cholesterol, Total | 1 | L – Creatinine | 3 |
| 4 | L – Cholesterol, LDL | 1 | L – WBC | 1 | L – Creatinine | 3 | L – Cholesterol, LDL Calc | 1 | P – BNF 6.1.2.1: Sulphonylureas | 1 |
| 5 | L – Hemoglobin A1c, IFCC | 1 | L – RBC | 1 | L – Potassium | 3 | L – Albumin, Urine, Spot | 1 | L – Sodium | 3 |
| 6 | L – BP (Systolic) | 1 | L – Platelet | 1 | D – ICPC K86: Hypertension uncomplicated | 1 | L – Cholesterol, LDL | 1 | L – Potassium | 3 |
| 7 | L – BP (Diastolic) | 1 | L – eGFR (MDRD) | 1 | L – eGFR (MDRD) | 1 | L – eGFR (MDRD) | 1 | L – Albumin, Urine, Spot | 1 |
| 8 | D – ICPC K86: Hypertension uncomplicated | 1 | L – Haemoglobin, Blood | 1 | D – ICD-9 250: Diabetes mellitus | 1 | L – Fasting Blood Sugar | 1 | L – eGFR (MDRD) | 1 |
| 9 | D – ICD-9 434: Occlusion of cerebral arteries | 1 | L – Cholesterol, LDL | 1 | D – ICPC T90: Diabetes non-insulin dependent | 2 | L – Haemoglobin A1C | 1 | L – eGFR (CKD-EPI) | 1 |
| 10 | P – BNF 2.9: Antiplatelet drugs | 1 | D – ICD-9 434: Occlusion of cerebral arteries | 1 | P – BNF 6.1.2.1: Sulphonylureas | 3 | L – Glucose, Random | 1 | L – Haemoglobin, Blood | 3 |
| 11 | L – Cholesterol, HDL | 1 | L – Albumin, Urine, Spot | 1 | L – Albumin, Urine, Spot | 1 | L – Fasting Blood Sugar | 1 | L – Platelet | 3 |
| 12 | L – Triglycerides | 1 | L – Fasting Blood Sugar | 1 | L – Fasting Blood Sugar | 1 | L – Glucose, Fasting | 1 | L – WBC | 3 |
| 13 | L – Cholesterol, Total | 1 | D – ICPC K86: Hypertension uncomplicated | 1 | D – ICD-9 434: Occlusion of cerebral arteries | 1 | D – ICPC T90: Diabetes non-insulin dependent | 1 | L – RBC | 3 |
| 14 | L – Creatine Kinase | 1 | L – Basophil, absolute | 1 | L – Cholesterol, Total | 1 | L – Calcium | 1 | L – Albumin | 3 |
| 15 | L – Cholesterol, LDL Calc | 1 | L – Neutrophil, absolute | 1 | L – Triglycerides | 1 | L – Platelet | 2 | L – Bilirubin, Total | 3 |
| 16 | D – ICPC T93: Lipid disorder | 1 | L – Lymphocyte, absolute | 1 | L – Cholesterol, HDL | 1 | L – WBC | 2 | L – Alkaline Phosphatase, Total | 3 |
| 17 | L – Alanine Aminotransferase | 1 | L – Neutrophil, % | 1 | L – Cholesterol, LDL | 1 | L – RBC | 2 | L – BP (Systolic) | 2 |
| 18 | L – Troponin I | 1 | L – Monocyte, absolute | 1 | L – Creatine Kinase | 1 | L – Haemoglobin, Blood | 2 | L – BP (Diastolic) | 2 |
| 19 | P – BNF 2.9: Antiplatelet drugs | 2 | L – Eosinophil, absolute | 1 | L – Albumin, Urine, Creatinine Ratio | 1 | P – BNF 6.1.2.2: Biguanides | 1 | L – Glucose, Random | 1 |
| 20 | L – eGFR (CKD-EPI) | 3 | L – Calcium | 1 | L – Cholesterol, LDL Calc | 1 | L – WBC | 1 | L – Alanine Aminotransferase | 3 |

Abbreviations: ACEI = Angiotensin-Converting Enzyme Inhibitor, ARB = Angiotensin-II Receptor Blocker, CCB = Calcium Channel Blocker

R column = Recurrence cut-off: 1 = One, 2 = Median, 3 = Third quartile

L = Laboratory measurement, D = Diagnosis, P = Prescription, eGFR = estimated Glomerular Filtration Rate, CKD-EPI = Chronic Kidney Disease Epidemiology Collaboration, MDRD = Modification of Diet in Renal Disease, HDL = High-Density Lipoprotein, LDL = Low-Density Lipoprotein, IFCC = International Federation of Clinical Chemistry, ICPC = International Classification of Primary Care code, ICD = International Classification of Diseases code, BNF = British National Formulary chapter, LDL Calc = LDL calculated based on total and HDL cholesterol and triglycerides, WBC = White Blood Cell count, RBC = Red Blood Cell count, BP = Blood Pressure, HDPS = High-dimensional Propensity Score

Supplementary Table 4. List of top 3 Bross-ranked high-dimensional propensity score covariates for each antihypertensive class comparison that are empirically strongly related with exposure but not the outcome*

|  | **Comparison vs ACEI** | | | | | | | | | |
| --- | --- | --- | --- | --- | --- | --- | --- | --- | --- | --- |
| **Top 3 HDPS covariates that behave like IVs** | **ARB** | | **Beta-blockers** | | **CCB** | | **Diuretics** | | **Combination** | |
|  | **Covariate** | **R** | **Covariate** | **R** | **Covariate** | **R** | **Covariate** | **R** | **Covariate** | **R** |
| 1 | P – BNF 6.1.2.3: Other antidiabetic drugs | 1 | P – BNF 6.1.2.1: Sulphonylureas | 1 | P – BNF 6.1.2.1: Sulphonylureas | 1 | D – ICPC T90: Diabetes non-insulin dependent | 1 | D – ICPC T90: Diabetes non-insulin dependent | 2 |
| 2 | N/A |  | L – Albumin, Urine, Spot | 1 | D – ICPC T90: Diabetes non-insulin dependent | 2 | P – BNF 6.1.2.2: Biguanides | 1 | D – ICPC T93: Lipid disorder | 1 |
| 3 | N/A |  | L – Albumin, Urine, Creatinine Ratio | 1 | D – ICPC T90: Diabetes non-insulin dependent | 3 | L – INR | 3 | D – ICPC T90: Diabetes non-insulin dependent | 3 |

Abbreviations: ACEI = Angiotensin-Converting Enzyme Inhibitor, ARB = Angiotensin-II Receptor Blocker, CCB = Calcium Channel Blocker, HDPS = High-dimensional Propensity Score, IV= instrumental variable

R column = Recurrence cut-off: 1 = One, 2 = Median, 3 = Third quartile

L = Laboratory measurement, D = Diagnosis, P = Prescription, BNF = British National Formulary chapter, ICPC = International Classification of Primary Care code, INR = International Normalized Ratio

* |logRR_CE_| > 1.5 and |logRR_CD_| < 0.5 where RR_CE_ is the risk ratio for covariate-exposure and RR_CD_ is the risk ratio for covariate-outcome

Supplementary Table 5. Sensitivity analysis: Exploring potentially influential high-dimensional propensity score covariates

| **Class** | IPTW, Adjusted HR (95% CI) | HDPS IPTW, Adjusted HR  (95% CI)  (250 covariates) | HDPS IPTW with top 10 Bross-ranked covariates only  (10 covariates) | HDPS IPTW removing covariates empirically strongly related with the exposure but not the outcome* | |
| --- | --- | --- | --- | --- | --- |
|  |  |  |  | Covariates removed** | Adjusted HR  (95% CI) |
| ACEI | Ref. | Ref. | Ref. |  | Ref. |
| ARB | 1.05 (0.79-1.39) | 1.00 (0.77-1.31) | 1.02 (0.79-1.31) | 1 | 1.01 (0.77-1.31) |
| Beta-blockers | 0.93 (0.86-1.02) | 0.90 (0.82-0.98) | 0.91 (0.84-0.98) | 27 | 0.90 (0.83-0.98) |
| CCB | 0.96 (0.90-1.03) | 0.96 (0.90-1.03) | 0.98 (0.92-1.05) | 13 | 0.96 (0.90-1.03) |
| Diuretics | 1.00 (0.91-1.10) | 0.94 (0.85-1.03) | 0.91 (0.83-1.00) | 46 | 0.94 (0.85-1.03) |
| Combination | 0.94 (0.83-1.07) | 1.00 (0.87-1.16) | 0.96 (0.84-1.10) | 16 | 1.00 (0.87-1.16) |

Abbreviations: IPTW = Inverse Probability of Treatment Weighting, HR = Hazard Ratio, CI = Confidence Interval, ACEI = Angiotensin-Converting Enzyme Inhibitor, ARB = Angiotensin-II Receptor Blocker, CCB = Calcium Channel Blocker

* |logRR_CE_| > 1.5 and |logRR_CD_| < 0.5 where RR_CE_ is the risk ratio for covariate and exposure, and RR_CD_ is the risk ratio for covariate and outcome.

** Covariates removed from top 250 HDPS covariates.
