# Supplementary material for: High‐Dimensional Propensity Scores for Mitigating Confounding: Implementation Using Primary and Secondary Care Data in Hong Kong
Source: Pharmacoepidemiol Drug Saf. 2026 Jan 25;35(2):e70326. doi: 10.1002/pds.70326 (PMC12833473; doi:10.1002/pds.70326)
Supplement: Supplementary file 2 — Data S1: pds70326‐sup‐0002‐Supinfo2.gz. [file PDS-35-e70326-s002.gz › hdps/inst/doc/hdps-tutorial.html]

HDPS Package Tutorial


# HDPS Package Tutorial

#### Min Fan, Edmund Chueng

#### 2025-10-24

# Introduction

The `hdps` package implements the High-Dimensional
Propensity Score (HDPS) algorithm for automated covariate selection in
observational studies.

## Key Features

- **Modular workflow**: 3-step process (identify → assess
  → prioritize)
- **Performance**: Parallel processing for large
  datasets
- **Visualizations**: Interactive plots for bias
  analysis
- **Data flexibility**: Multiple input formats

# Installation

```
# Install from GitHub
devtools::install_github("Cainefm/hdps")

# Or install from local .tar.gz file
install.packages("hdps_0.9.0.tar.gz", repos = NULL, type = "source")
```

# Basic Usage

## Load the Package

```
library(hdps)
library(data.table)
library(ggplot2)
```

## Example Dataset

Let’s create a sample dataset to demonstrate the package
functionality:

```
# Create sample diagnosis data
set.seed(123)
n_patients <- 100
n_codes <- 50

# Generate patient IDs and diagnosis codes
dx_data <- data.table(
  pid = rep(1:n_patients, each = n_codes),
  code = rep(paste0("ICD", sprintf("%03d", 1:n_codes)), n_patients),
  type = "dx"
)

# Add some variation - not all patients have all codes
dx_data <- dx_data[sample(nrow(dx_data), nrow(dx_data) * 0.3), ]

# Create master table with exposure and outcome
master_data <- data.table(
  pid = 1:n_patients,
  exposure = rbinom(n_patients, 1, 0.5),
  outcome = rbinom(n_patients, 1, 0.3)
)

# Merge with exposure/outcome data
dx_data <- merge(dx_data, master_data, by = "pid", all.x = TRUE)

head(dx_data)
```

```
## Key: <pid>
##      pid   code   type exposure outcome
##    <int> <char> <char>    <int>   <int>
## 1:     1 ICD041     dx        1       0
## 2:     1 ICD031     dx        1       0
## 3:     1 ICD049     dx        1       0
## 4:     1 ICD044     dx        1       0
## 5:     1 ICD045     dx        1       0
## 6:     1 ICD001     dx        1       0
```

## Step 1: Identify Candidate Covariates

```
# Identify candidate covariates
candidates <- identify_candidates(
  dx_data, 
  id = "pid", 
  code = "code", 
  type = "dx",
  n = 20,  # Maximum 20 candidates
  min_patients = 5  # Minimum 5 patients per code
)

# View candidates
head(candidates$candidates)
```

```
##      code n_patients prevalence prevalence_truncated
##    <char>      <int>      <num>                <num>
## 1: ICD049         43         43                   43
## 2: ICD006         41         41                   41
## 3: ICD036         38         38                   38
## 4: ICD015         37         37                   37
## 5: ICD005         37         37                   37
## 6: ICD004         37         37                   37
```

## Step 2: Assess Recurrence

```
# Assess recurrence patterns
recurrence <- assess_recurrence(
  candidates$data, 
  id = "pid", 
  code = "code", 
  type = "dx"
)

# View recurrence data
head(recurrence)
```

```
## Key: <pid>
##      pid dx_dx_ICD001_once dx_dx_ICD003_once dx_dx_ICD004_once
##    <int>             <num>             <num>             <num>
## 1:     1                 1                 0                 0
## 2:     2                 0                 0                 0
## 3:     3                 0                 0                 0
## 4:     4                 1                 0                 1
## 5:     5                 1                 0                 1
## 6:     6                 1                 0                 0
##    dx_dx_ICD005_once dx_dx_ICD006_once dx_dx_ICD008_once dx_dx_ICD009_once
##                <num>             <num>             <num>             <num>
## 1:                 0                 0                 0                 0
## 2:                 0                 1                 0                 0
## 3:                 0                 0                 0                 0
## 4:                 1                 0                 0                 0
## 5:                 0                 0                 0                 0
## 6:                 0                 1                 1                 1
##    dx_dx_ICD011_once dx_dx_ICD015_once dx_dx_ICD020_once dx_dx_ICD022_once
##                <num>             <num>             <num>             <num>
## 1:                 1                 0                 0                 0
## 2:                 0                 0                 1                 0
## 3:                 0                 0                 1                 0
## 4:                 1                 1                 0                 0
## 5:                 0                 0                 1                 0
## 6:                 0                 0                 1                 0
##    dx_dx_ICD026_once dx_dx_ICD027_once dx_dx_ICD031_once dx_dx_ICD036_once
##                <num>             <num>             <num>             <num>
## 1:                 0                 1                 1                 1
## 2:                 1                 1                 0                 0
## 3:                 0                 0                 0                 0
## 4:                 0                 0                 0                 0
## 5:                 0                 0                 0                 0
## 6:                 1                 1                 0                 0
##    dx_dx_ICD037_once dx_dx_ICD039_once dx_dx_ICD041_once dx_dx_ICD042_once
##                <num>             <num>             <num>             <num>
## 1:                 1                 0                 1                 0
## 2:                 0                 0                 0                 0
## 3:                 1                 1                 1                 0
## 4:                 0                 0                 1                 0
## 5:                 0                 0                 1                 1
## 6:                 1                 0                 1                 0
##    dx_dx_ICD049_once
##                <num>
## 1:                 1
## 2:                 0
## 3:                 0
## 4:                 0
## 5:                 1
## 6:                 1
```

## Step 3: Prioritize Covariates

```
# Merge with exposure/outcome data
cohort_data <- merge(recurrence, master_data, by = "pid", all.x = TRUE)

# Prioritize covariates
prioritization <- prioritize(
  cohort_data, 
  pid = "pid", 
  expo = "exposure", 
  outc = "outcome"
)

# View top covariates
head(prioritization[order(absLogBias, decreasing = TRUE)])
```

```
##                 code    e1    e0    d1    d0    c1    c0  e1c1  e0c1  e1c0
##               <char> <int> <int> <int> <int> <int> <int> <int> <int> <int>
## 1: dx_dx_ICD004_once    49    51    27    73    37    63    24    13    25
## 2: dx_dx_ICD003_once    49    51    27    73    35    65    14    21    35
## 3: dx_dx_ICD022_once    49    51    27    73    31    69    18    13    31
## 4: dx_dx_ICD039_once    49    51    27    73    31    69    21    10    28
## 5: dx_dx_ICD006_once    49    51    27    73    41    59    17    24    32
## 6: dx_dx_ICD037_once    49    51    27    73    36    64    15    21    34
##     e0c0  d1c1  d0c1  d1c0  d0c0       pc1       pc0      rrCE      rrCD
##    <int> <int> <int> <int> <int>     <num>     <num>     <num>     <num>
## 1:    38     7    30    20    43 0.4897959 0.2549020 1.9215071 0.5959459
## 2:    30     5    30    22    43 0.2857143 0.4117647 0.6938776 0.4220779
## 3:    38     4    27    23    46 0.3673469 0.2549020 1.4411303 0.3870968
## 4:    41    10    21    17    52 0.4285714 0.1960784 2.1857143 1.3092979
## 5:    27    14    27    13    46 0.3469388 0.4705882 0.7372449 1.5497186
## 6:    30     7    29    20    44 0.3061224 0.4117647 0.7434402 0.6222222
##         bias absLogBias ce_strength cd_strength
##        <num>      <num>       <num>       <num>
## 1: 0.8941926 0.11183409   0.9215071   0.4040541
## 2: 1.0955961 0.09129863   0.3061224   0.5779221
## 3: 0.9183215 0.08520778   0.4411303   0.6129032
## 4: 1.0677979 0.06559848   1.1857143   0.3092979
## 5: 0.9459975 0.05551530   0.2627551   0.5497186
## 6: 1.0472610 0.04617819   0.2565598   0.3777778
```

# Advanced Features

## Multi-Domain Analysis

```
# Create multi-domain data
dx_data <- data.table(pid = 1:100, code = paste0("DX", 1:100), type = "dx")
px_data <- data.table(pid = 1:100, code = paste0("PX", 1:100), type = "px")
rx_data <- data.table(pid = 1:100, code = paste0("RX", 1:100), type = "rx")

# Multi-domain analysis
multi_domain_results <- hdps_multi_domain(
  data_list = list(dx = dx_data, px = px_data, rx = rx_data),
  id_col = "pid",
  code_col = "code",
  exposure_col = "exposure",
  outcome_col = "outcome"
)
```

## Complete Workflow with hdps\_screen

```
# Complete workflow in one function
results <- hdps_screen(
  data = dx_data,
  id_col = "pid",
  code_col = "code",
  exposure_col = "exposure",
  outcome_col = "outcome",
  n_candidates = 20,
  min_patients = 5
)

# View results
str(results)
```

```
## List of 3
##  $ candidates    :List of 3
##   ..$ candidates :Classes 'data.table' and 'data.frame': 20 obs. of  4 variables:
##   .. ..$ code                : chr [1:20] "ICD049" "ICD006" "ICD036" "ICD015" ...
##   .. ..$ n_patients          : int [1:20] 43 41 38 37 37 37 37 36 36 35 ...
##   .. ..$ prevalence          : num [1:20] 43 41 38 37 37 37 37 36 36 35 ...
##   .. ..$ prevalence_truncated: num [1:20] 43 41 38 37 37 37 37 36 36 35 ...
##   .. ..- attr(*, ".internal.selfref")=<externalptr> 
##   ..$ data       :Classes 'data.table' and 'data.frame': 695 obs. of  5 variables:
##   .. ..$ pid     : int [1:695] 1 1 1 1 1 1 1 1 2 2 ...
##   .. ..$ code    : chr [1:695] "dx_ICD041" "dx_ICD031" "dx_ICD049" "dx_ICD001" ...
##   .. ..$ type    : chr [1:695] "dx" "dx" "dx" "dx" ...
##   .. ..$ exposure: int [1:695] 1 1 1 1 1 1 1 1 0 0 ...
##   .. ..$ outcome : int [1:695] 0 0 0 0 0 0 0 0 0 0 ...
##   .. ..- attr(*, ".internal.selfref")=<externalptr> 
##   .. ..- attr(*, "sorted")= chr "pid"
##   ..$ patient_ids: int [1:100] 1 2 3 4 5 6 7 8 9 10 ...
##  $ recurrence    :Classes 'data.table' and 'data.frame': 100 obs. of  21 variables:
##   ..$ pid               : int [1:100] 1 2 3 4 5 6 7 8 9 10 ...
##   ..$ cov_dx_ICD001_once: num [1:100] 1 0 0 1 1 1 0 1 0 0 ...
##   ..$ cov_dx_ICD003_once: num [1:100] 0 0 0 0 0 0 1 1 0 0 ...
##   ..$ cov_dx_ICD004_once: num [1:100] 0 0 0 1 1 0 1 1 0 0 ...
##   ..$ cov_dx_ICD005_once: num [1:100] 0 0 0 1 0 0 0 0 0 0 ...
##   ..$ cov_dx_ICD006_once: num [1:100] 0 1 0 0 0 1 0 0 1 0 ...
##   ..$ cov_dx_ICD008_once: num [1:100] 0 0 0 0 0 1 0 1 1 1 ...
##   ..$ cov_dx_ICD009_once: num [1:100] 0 0 0 0 0 1 0 0 1 1 ...
##   ..$ cov_dx_ICD011_once: num [1:100] 1 0 0 1 0 0 1 0 0 0 ...
##   ..$ cov_dx_ICD015_once: num [1:100] 0 0 0 1 0 0 0 0 1 0 ...
##   ..$ cov_dx_ICD020_once: num [1:100] 0 1 1 0 1 1 0 0 1 0 ...
##   ..$ cov_dx_ICD022_once: num [1:100] 0 0 0 0 0 0 1 1 1 0 ...
##   ..$ cov_dx_ICD026_once: num [1:100] 0 1 0 0 0 1 1 1 0 0 ...
##   ..$ cov_dx_ICD027_once: num [1:100] 1 1 0 0 0 1 0 0 0 0 ...
##   ..$ cov_dx_ICD031_once: num [1:100] 1 0 0 0 0 0 1 0 0 1 ...
##   ..$ cov_dx_ICD036_once: num [1:100] 1 0 0 0 0 0 0 1 1 0 ...
##   ..$ cov_dx_ICD037_once: num [1:100] 1 0 1 0 0 1 1 1 0 1 ...
##   ..$ cov_dx_ICD039_once: num [1:100] 0 0 1 0 0 0 0 0 0 0 ...
##   ..$ cov_dx_ICD041_once: num [1:100] 1 0 1 1 1 1 0 0 0 0 ...
##   ..$ cov_dx_ICD042_once: num [1:100] 0 0 0 0 1 0 0 0 0 1 ...
##   ..$ cov_dx_ICD049_once: num [1:100] 1 0 0 0 1 1 0 0 1 0 ...
##   ..- attr(*, ".internal.selfref")=<externalptr> 
##   ..- attr(*, "sorted")= chr "pid"
##  $ prioritization:Classes 'data.table' and 'data.frame': 20 obs. of  23 variables:
##   ..$ code       : chr [1:20] "cov_dx_ICD001_once" "cov_dx_ICD003_once" "cov_dx_ICD004_once" "cov_dx_ICD005_once" ...
##   ..$ e1         : int [1:20] 741 741 741 741 741 741 741 741 741 741 ...
##   ..$ e0         : int [1:20] 759 759 759 759 759 759 759 759 759 759 ...
##   ..$ d1         : int [1:20] 381 381 381 381 381 381 381 381 381 381 ...
##   ..$ d0         : int [1:20] 1119 1119 1119 1119 1119 1119 1119 1119 1119 1119 ...
##   ..$ c1         : int [1:20] 471 535 580 565 614 577 483 486 599 551 ...
##   ..$ c0         : int [1:20] 1029 965 920 935 886 923 1017 1014 901 949 ...
##   ..$ e1c1       : int [1:20] 264 211 379 292 255 314 256 241 319 216 ...
##   ..$ e0c1       : int [1:20] 207 324 201 273 359 263 227 245 280 335 ...
##   ..$ e1c0       : int [1:20] 477 530 362 449 486 427 485 500 422 525 ...
##   ..$ e0c0       : int [1:20] 552 435 558 486 400 496 532 514 479 424 ...
##   ..$ d1c1       : int [1:20] 116 76 102 107 200 160 134 131 134 125 ...
##   ..$ d0c1       : int [1:20] 355 459 478 458 414 417 349 355 465 426 ...
##   ..$ d1c0       : int [1:20] 265 305 279 274 181 221 247 250 247 256 ...
##   ..$ d0c0       : int [1:20] 764 660 641 661 705 702 770 764 654 693 ...
##   ..$ pc1        : num [1:20] 0.356 0.285 0.511 0.394 0.344 ...
##   ..$ pc0        : num [1:20] 0.273 0.427 0.265 0.36 0.473 ...
##   ..$ rrCE       : num [1:20] 1.306 0.667 1.931 1.096 0.728 ...
##   ..$ rrCD       : num [1:20] 0.956 0.449 0.58 0.646 1.594 ...
##   ..$ bias       : num [1:20] 0.996 1.102 0.883 0.986 0.94 ...
##   ..$ absLogBias : num [1:20] 0.0037 0.0974 0.124 0.014 0.0617 ...
##   ..$ ce_strength: num [1:20] 0.3063 0.3329 0.9314 0.0956 0.2724 ...
##   ..$ cd_strength: num [1:20] 0.0437 0.5505 0.4201 0.3538 0.5945 ...
##   ..- attr(*, ".internal.selfref")=<externalptr>
```

# Visualization

## Bias Distribution Plot

```
# Plot bias distribution
p1 <- plot_bias_distribution(prioritization, top_n = 10)
print(p1)
```

## Covariate Strength Relationships

```
# Plot covariate strength relationships
p2 <- plot_covariate_strength(prioritization)
print(p2)
```

## Bias vs Prevalence

```
# Plot bias vs prevalence
p3 <- plot_bias_vs_prevalence(prioritization)
print(p3)
```

## Interactive Plots

```
# Create interactive plots
library(plotly)

# Interactive bias distribution
p1_interactive <- plot_bias_distribution(prioritization, top_n = 10, interactive = TRUE)
p1_interactive

# Interactive strength plot
p2_interactive <- plot_covariate_strength(prioritization, interactive = TRUE)
p2_interactive
```

# Flexible Data Input

## Different Input Formats

```
# Long format (default)
data_long <- hdps_input(dx_data, format = "long")

# Wide format
wide_data <- dcast(dx_data, pid ~ code, value.var = "code", fun.aggregate = length)
data_wide <- hdps_input(wide_data, format = "wide", value_col = "count")

# Matrix format (example)
# matrix_data <- matrix(rbinom(100, 1, 0.5), nrow = 10, ncol = 10)
# rownames(matrix_data) <- paste0("P", 1:10)
# colnames(matrix_data) <- paste0("VAR", 1:10)
# data_matrix <- hdps_input(matrix_data, format = "matrix")
```

# Performance Tips

## Large Datasets

For large datasets, consider:

1. **Limit candidates**: Use smaller `n` values
   in `identify_candidates()`
2. **Filter data**: Remove rare codes before analysis
3. **Use data.table**: Ensure your data is in
   `data.table` format

## Memory Management

```
# Monitor memory usage
library(pryr)
mem_used()

# Use gc() to free memory
gc()
```

# Best Practices

## Data Preparation

1. **Clean your data**: Remove missing values and invalid
   codes
2. **Standardize formats**: Ensure consistent column names
   and data types
3. **Validate inputs**: Check that required columns
   exist

## Parameter Selection

1. **n\_candidates**: Start with 200-500, adjust based on
   your data size
2. **min\_patients**: Use 10-50 depending on your sample
   size

## Quality Control

```
# Check data quality
cat("Number of patients:", length(unique(dx_data$pid)), "\n")
```

```
## Number of patients: 100
```

```
cat("Number of codes:", length(unique(dx_data$code)), "\n")
```

```
## Number of codes: 50
```

```
cat("Missing values:", sum(is.na(dx_data)), "\n")
```

```
## Missing values: 0
```

```
# Check exposure/outcome distribution
table(master_data$exposure)
```

```
## 
##  0  1 
## 51 49
```

```
table(master_data$outcome)
```

```
## 
##  0  1 
## 73 27
```

# Troubleshooting

## Common Issues

1. **Memory errors**: Reduce `n_candidates` or
   filter data
2. **Slow performance**: Use data.table for better
   performance
3. **Missing data**: Check for NA values in your
   dataset
4. **Column name errors**: Ensure column names match
   exactly

## Debugging

```
# Check intermediate results
candidates <- identify_candidates(dx_data, "pid", "code", "dx", n = 10)
cat("Number of candidates:", nrow(candidates$candidates), "\n")
```

```
## Number of candidates: 10
```

```
# Check recurrence data
recurrence <- assess_recurrence(candidates$data, "pid", "code", "dx")
cat("Number of recurrence variables:", ncol(recurrence) - 1, "\n")
```

```
## Number of recurrence variables: 10
```

# Conclusion

The `hdps` package provides a comprehensive solution for
high-dimensional propensity score analysis. Key features include:

- **Modular workflow** for flexible analysis
- **Domain-specific handling** for different data
  types
- **Parallel processing** for large datasets
- **Enhanced visualizations** for result
  interpretation
- **Flexible data input** for various formats

For more information, see the package documentation and examples.
