# Supplementary figures and images for: High‐Dimensional Propensity Scores for Mitigating Confounding: Implementation Using Primary and Secondary Care Data in Hong Kong
Source: Pharmacoepidemiol Drug Saf. 2026 Jan 25;35(2):e70326. doi: 10.1002/pds.70326 (PMC12833473; doi:10.1002/pds.70326)

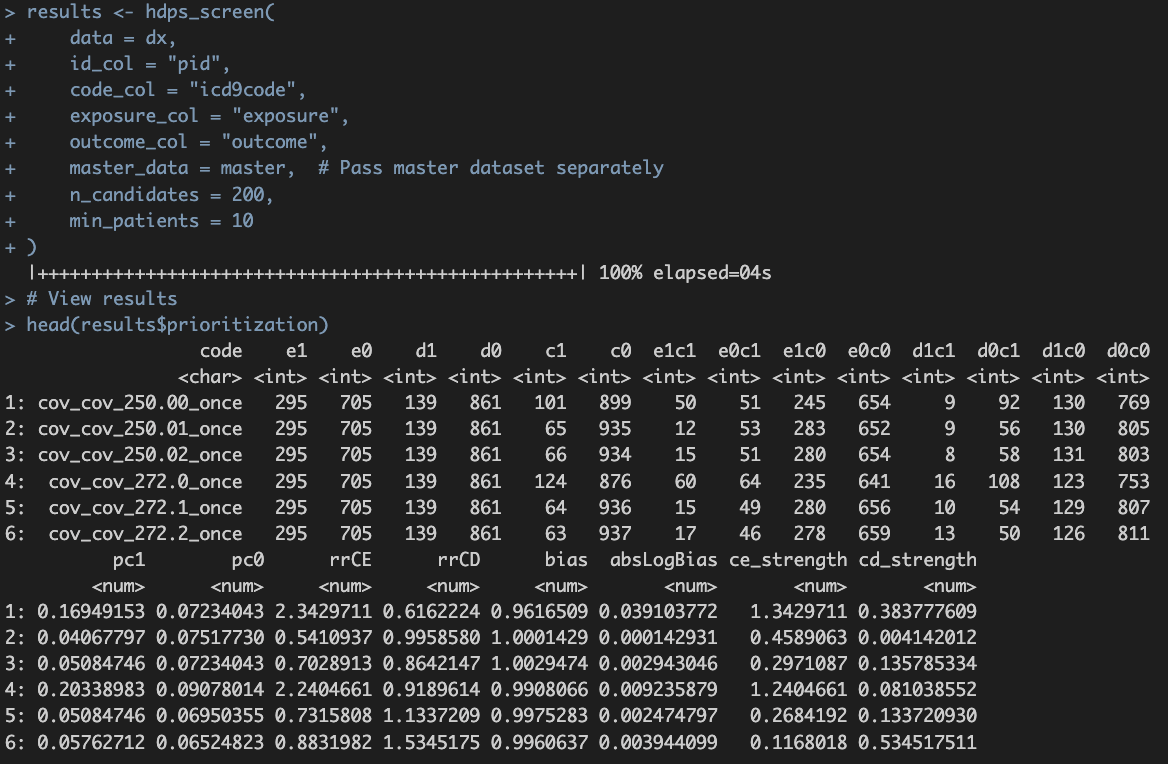

Supplement: Supplementary file 2 — Data S1: pds70326‐sup‐0002‐Supinfo2.gz. [file PDS-35-e70326-s002.gz › hdps/data/Snipaste_2025-10-24_17-07-48.png]

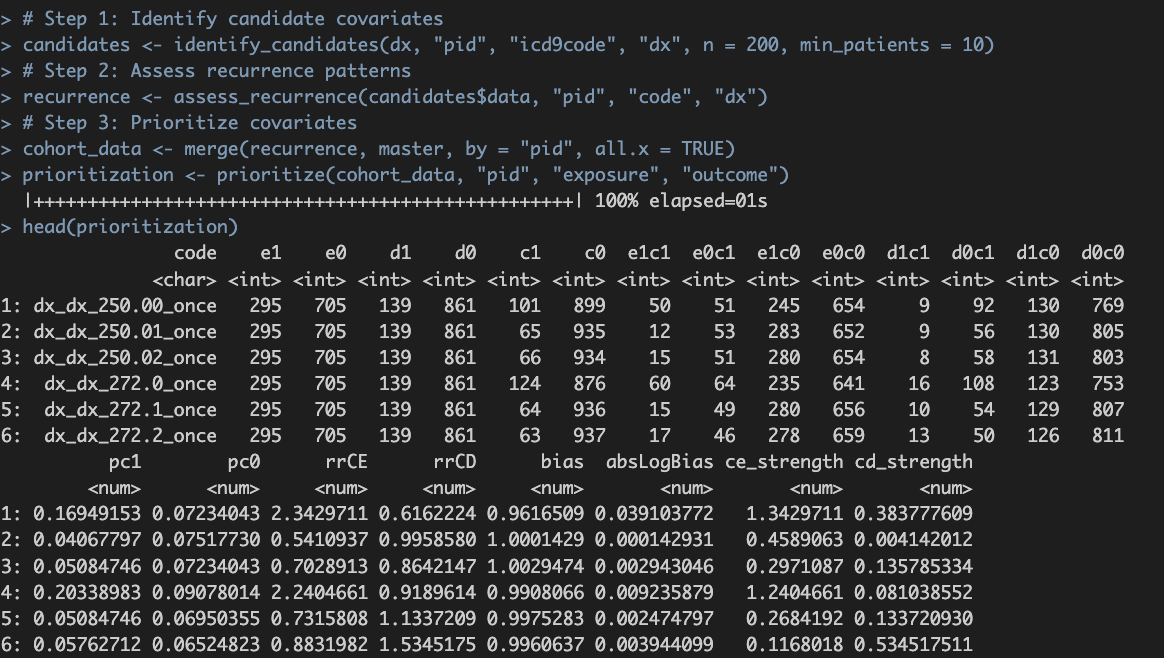

Supplement: Supplementary file 2 — Data S1: pds70326‐sup‐0002‐Supinfo2.gz. [file PDS-35-e70326-s002.gz › hdps/data/Snipaste_2025-10-24_17-09-12.png]

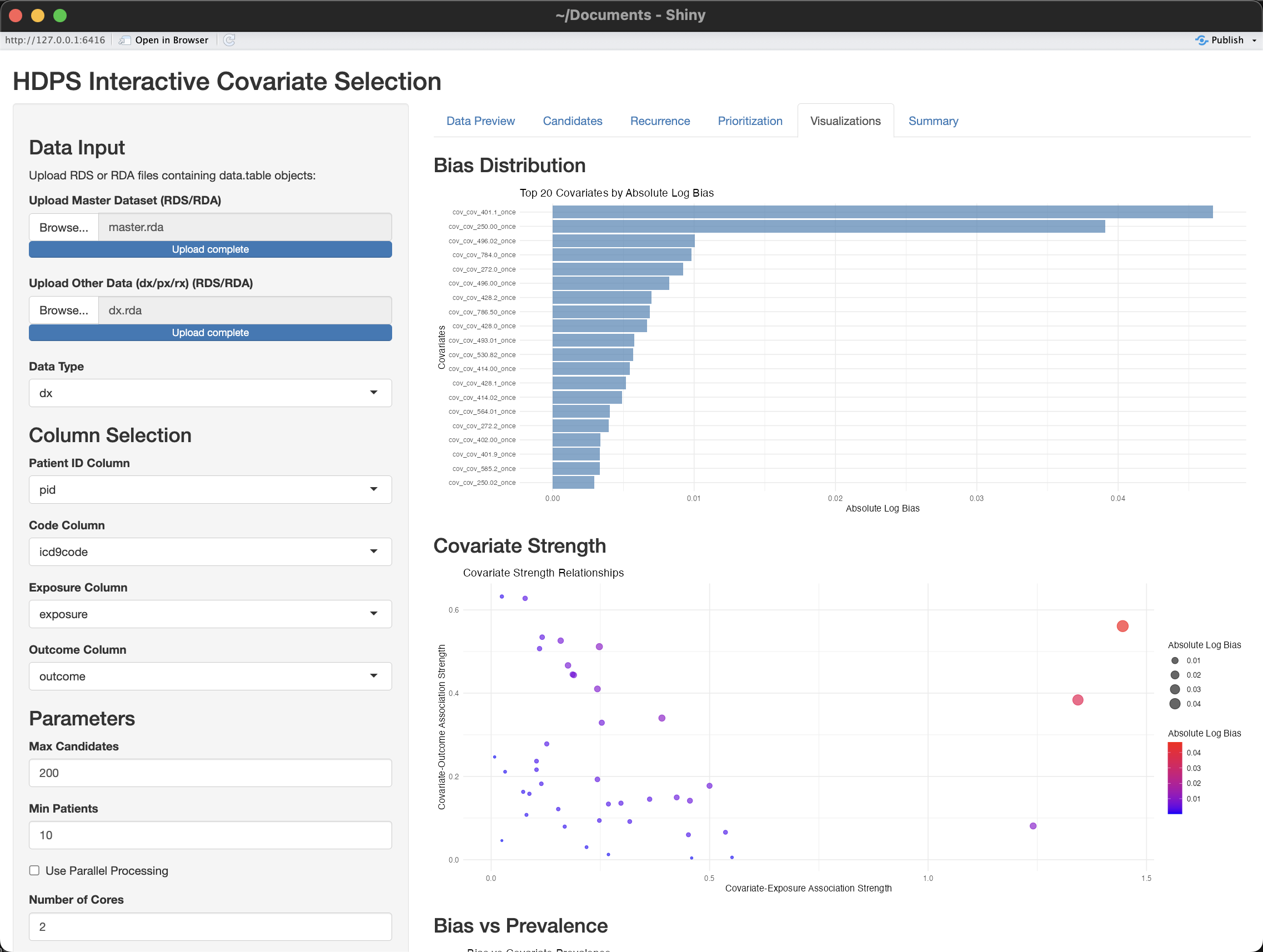

Supplement: Supplementary file 2 — Data S1: pds70326‐sup‐0002‐Supinfo2.gz. [file PDS-35-e70326-s002.gz › hdps/data/Snipaste_2025-10-24_17-10-32.png]
